# Correction: First Clarkforkian Equivalent Land Mammal Age in the Latest Paleocene Basal Sparnacian Facies of Europe: Fauna, Flora, Paleoenvironment and (Bio)stratigraphy

**DOI:** 10.1371/journal.pone.0093249

**Published:** 2014-03-25

**Authors:** The PLOS ONE Staff

## Notice of Republication

This article was republished on February 27, 2014, to replace an incorrectly published version in which Figures 4, 6, and 7 were erroneously re-sized in the PDF. Please download this article again to view the correct version. The originally published, uncorrected article and the republished, corrected article are provided here for reference.

## Supporting Information

File S1 Originally published, uncorrected article

File S2 Republished, corrected article
