# Supplementary figures and images for: First Clarkforkian Equivalent Land Mammal Age in the Latest Paleocene Basal Sparnacian Facies of Europe: Fauna, Flora, Paleoenvironment and (Bio)stratigraphy
Source: PLoS One. 2014 Jan 29;9(1):e86229. doi: 10.1371/journal.pone.0086229 (PMC3906055; doi:10.1371/journal.pone.0086229)

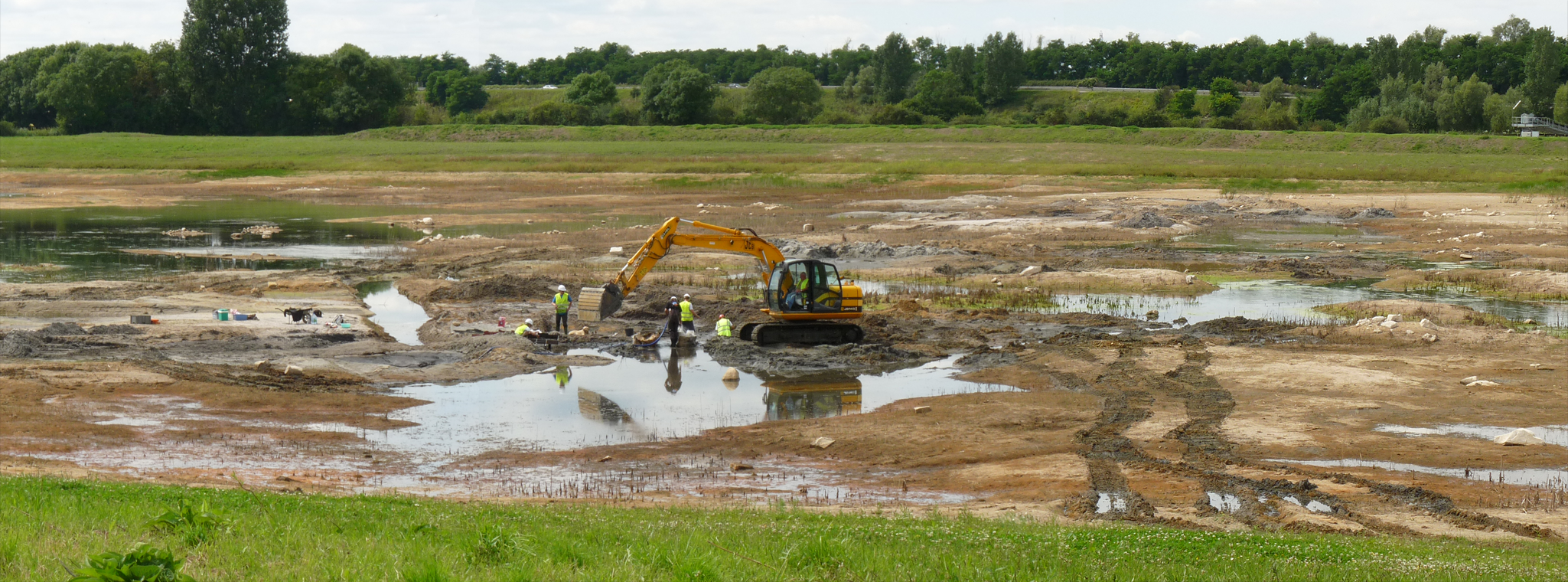

Supplement: Figure S1 — Fieldwork in central part of the Petit Pâtis Quarry in Rivecourt (summer 2012). North is at right, south at left and the RD200 road on the backside. The mechanical shovel is just behind section Rive 2. (TIF) [file pone.0086229.s001.tif]

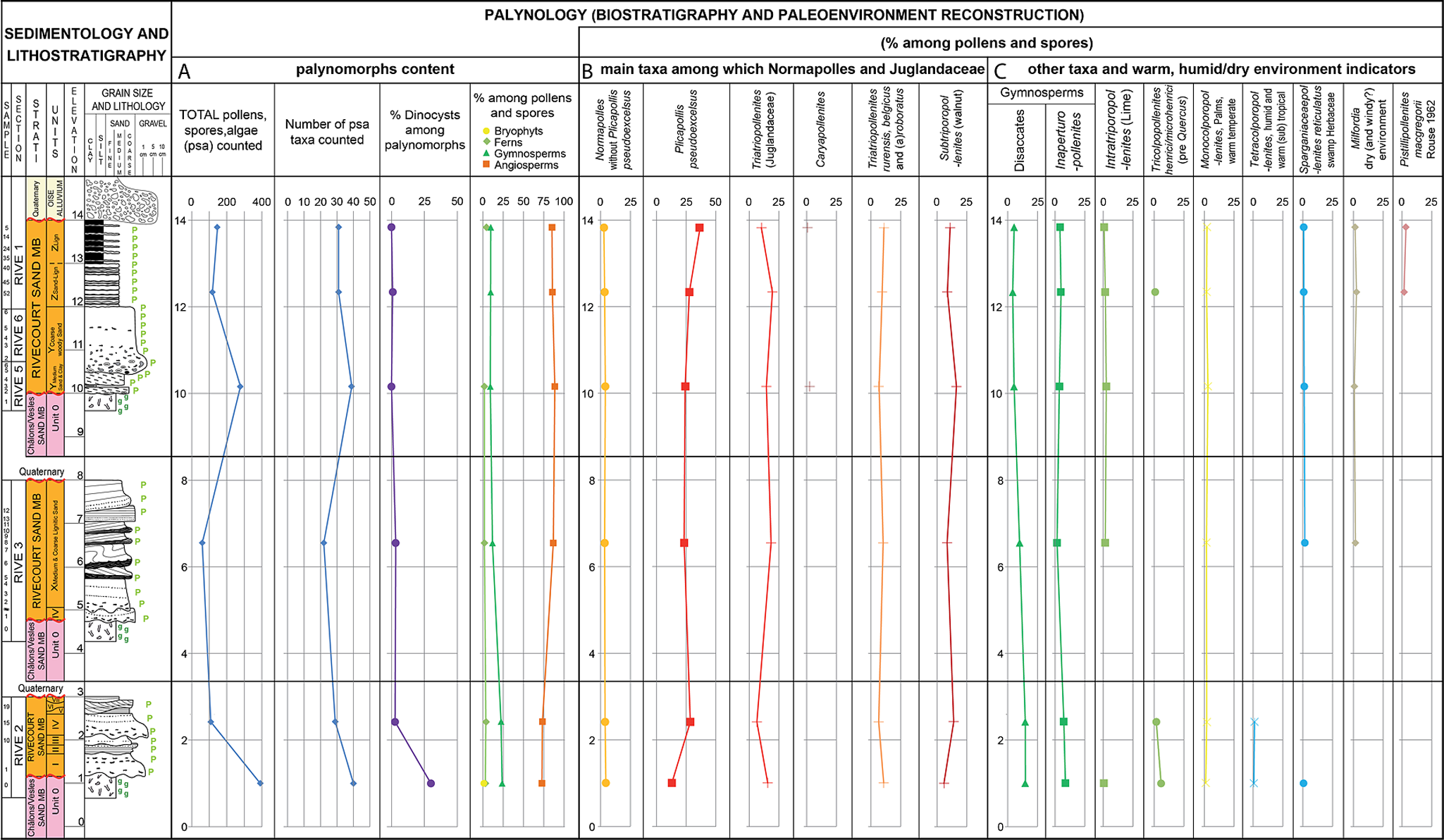

Supplement: Figure S2 — Palynology of the Petit Pâtis Quarry in Rivecourt. Sedimentologic log of the composite section (same legend as for Fig. 3) and palynology abundance curves. (A) Palynomorphs content, (B) Main pollen taxa among which Normapolles and Juglandaceae. (C) Gymnosperms, lime and pre-Quercus pollen grains and warm, humid/dry environment indicators. (TIF) [file pone.0086229.s002.tif]
