# Supplementary material for: First Clarkforkian Equivalent Land Mammal Age in the Latest Paleocene Basal Sparnacian Facies of Europe: Fauna, Flora, Paleoenvironment and (Bio)stratigraphy
Source: PLoS One. 2014 Jan 29;9(1):e86229. doi: 10.1371/journal.pone.0086229 (PMC3906055; doi:10.1371/journal.pone.0086229)
Supplement: Table S1 — Sedimentological (paleocurrents) compass measurements on the Petit Pâtis Quarry in Rivecourt. (A) Direction and angle of dip of the cross-beds from the RIVE 2 and RIVE 3 subsections; (B) Direction and size of the wood debris from the RIVE 2 subsection. (DOC) [file pone.0086229.s003.doc]

| **A** | | | | **B** | | | | |
| --- | --- | --- | --- | --- | --- | --- | --- | --- |
| Subsection | Unit | Cross-beds | | Subsection | Unit | Wood debris | | |
|  |  | Direction of Dip (°N) | Angle of Dip (°) |  |  | length (cm) | diameter (cm) | direction (°N) |
| RIVE 3 | Xe | 205 | 16 | RIVE 2 | IV | 50 | 4 | 102 |
|  | Xd | 205 | 7 |  |  | 15 | 7 | 42 |
|  |  | 205 | 10 |  |  | 17 | 5 | 82 |
| RIVE 2 | VI | 280 | 10 |  |  | 30 | 11 | 33 |
|  |  | 280 | 20 |  |  | 80 | 6 | 66 |
|  |  | 285 | 16 |  | I | 10 | 5 | 48 |
|  | V | 270 | 17 |  |  | 25 | 10 | 52 |
|  |  | 270 | 30 |  |  |  |  |  |
|  | III | 265 | 16 |  |  |  |  |  |
|  | II | 265 | 11 |  |  |  |  |  |
|  | I | 265 | 24 |  |  |  |  |  |
