# Supplementary material for: First Clarkforkian Equivalent Land Mammal Age in the Latest Paleocene Basal Sparnacian Facies of Europe: Fauna, Flora, Paleoenvironment and (Bio)stratigraphy
Source: PLoS One. 2014 Jan 29;9(1):e86229. doi: 10.1371/journal.pone.0086229 (PMC3906055; doi:10.1371/journal.pone.0086229)
Supplement: Table S2 — Chemostratigraphic data of the Petit Pâtis Quarry composite section in Rivecourt. Lithology, elevation (m), samples number, TOC (%) and δ13Corg values (average of 2 measurements) of all the samples analyzed. (DOC) [file pone.0086229.s004.doc]

| **Lithology** | **Composite log elevation (m)** | **Sample Number** | **TOC (%)** | **δ^13^C_org_ (% average)** |
| --- | --- | --- | --- | --- |
| Lignite | 13.84 | RIVE 1 - 5 | 27.90 | -26.0 |
| Lignite | 13.61 | RIVE 1 - 14 | 32.20 | -26.0 |
| Laminated lignite | 13.36 | RIVE 1 - 24 | 22.90 | -26.1 |
| Laminated lignite | 13.06 | RIVE 1 - 35 | 34.70 | -25.9 |
| Lignite | 12.90 | RIVE 1 - 40 | 25.10 | -26.4 |
| Lignitic sand | 12.58 | RIVE 1 - 45 | 1.96 | -25.3 |
| Lignite | 12.34 | RIVE 1 - 52 | 15.50 | -26.3 |
| Laminated clay | 10.16 | RIVE 5 - 3 | 1.54 | -26.6 |
| Coarse sand, with quartz, flint and feldspar grains | 7.10 | RIVE 3 - 13 | 0.08 | -24.9 |
| Medium-coarse sand, very rich in black vegetal debris, charcoal pebbles and flower, seed and fruit pyritized fossils | 6.55 | RIVE 3 - 8 | 15.20 | -24.9 |
| Medium sand, very rich in organic matter and black vegetal debris | 6.08 | RIVE 3 - 6 | 6.53 | -26.6 |
| Isolated charcoal pebble | 5.00 | CHAB, base X | 75.20 | -24.4 |
| Very coarse sand, poorly sorted, silty and organic, with fauna, flora and flint grains and centimetric flint pebbles | 4.85 | RIVE 4 - 1 | 0.18 | -24.5 |
| Medium sand, very rich in organic matter and black vegetal debris | 2.78 | RIVE 2 - 19 | 19.70 | -25.1 |
| Coarse to medium sand, with wood debris, vertebrate fauna, pyritized molluscs moulds, rounded flint gravels | 2.40 | RIVE 2 - 15 | 0.36 | -25.7 |
| Medium sand, well sorted, dispersed organic matter | 1.98 | RIVE 2 - 10 | 0.27 | -26.6 |
| Coarse to medium sand, well sorted, not very rich in organic matter | 1.32 | RIVE 2 - 1 | 0.06 | -26.2 |
