# Supplementary material for: First Clarkforkian Equivalent Land Mammal Age in the Latest Paleocene Basal Sparnacian Facies of Europe: Fauna, Flora, Paleoenvironment and (Bio)stratigraphy
Source: PLoS One. 2014 Jan 29;9(1):e86229. doi: 10.1371/journal.pone.0086229 (PMC3906055; doi:10.1371/journal.pone.0086229)
Supplement: Table S3 — Diversity of dinoflagellate cysts of the Petit Pâtis Quarry composite section in Rivecourt. (DOC) [file pone.0086229.s005.doc]

| **Lithostratigraphy** | **Unit 0** | | **Unit IV** | | **Unit x** | | **Unit y** | | **Unit z (Lignite)** | | | |  |
| --- | --- | --- | --- | --- | --- | --- | --- | --- | --- | --- | --- | --- | --- |
| **Elevation in the composite section (m)** | 0.9 | | 2.4 | | 6.55 | | 10.1 | | 12.33 | | 13.84 | |  |
| **SLIDE LABEL** | MPA 59942 | | MPA 59941 | | TGK 7 | | TGK 8 | | TGK 6 | | TGK 5 | |  |
| **SAMPLE** | **RIVE 2-0** | | **RIVE 2-15** | | **RIVE 3-8** | | **RIVE 5-3** | | **RIVE 1-52** | | **RIVE 1-5** | |  |
| **Prasinophytes** |  |  | |  | |  | |  | |  | |  | |
| *Pterospermella aureolata* | 2 |  | |  | |  | |  | |  | |  | |
| *Pterospermella barbarae* | 3 |  | |  | |  | |  | |  | |  | |
| **Acritarchs** |  |  | |  | |  | |  | |  | |  | |
| *Micrhystridium spp.* | 32 |  | |  | |  | |  | |  | |  | |
| *Paucilibimorpha incurvata* | 1 |  | |  | |  | |  | |  | |  | |
| **Dinoflagellate cysts** |  |  | |  | |  | |  | |  | |  | |
| *Achomosphaera alcicornu* | 0 |  | |  | |  | |  | |  | |  | |
| *Adnatosphaeridium* spp. (broaken) | 0 |  | |  | |  | |  | |  | |  | |
| *Alisocysta margarita* | 1 |  | |  | |  | |  | |  | |  | |
| *Areoligera coronata* | 8 |  | |  | |  | |  | |  | |  | |
| *Cerodinium* or *Deflandrea* (broaken) | 1 |  | |  | |  | |  | |  | |  | |
| *Cerodinium* spp. | 1 |  | |  | |  | |  | |  | |  | |
| *Cordosphaeridium inodes* | 0 |  | |  | |  | |  | |  | |  | |
| *Cordosphaeridium* spp. | 2,5 |  | |  | |  | |  | |  | |  | |
| *Cribroperidinium tenuitabulatum* | 1 |  | |  | |  | |  | |  | |  | |
| *Dapsilidinium pseudocolligerum* | 3 |  | |  | |  | |  | |  | |  | |
| *Diphyes colligerum* | 4 |  | |  | |  | |  | |  | |  | |
| *Elitrocysta brevis* | 2 |  | |  | |  | |  | |  | |  | |
| *Fibrocysta vectensis* | 5 |  | |  | |  | |  | |  | |  | |
| *Glaphyrocysta ordinata* | 0 | 1 | |  | |  | |  | |  | |  | |
| *Glaphyrocysta* spp. | 0 | 1 | |  | |  | |  | |  | |  | |
| *Hafniasphaera septata* | 2 |  | |  | |  | |  | |  | |  | |
| *Hystrichokolpoma rigaudiae* | 1 |  | |  | |  | |  | |  | |  | |
| *Hystrichokolpoma salacium* | 0 |  | |  | |  | |  | |  | |  | |
| *Hystrichokolpoma* spp. | 2 |  | |  | |  | |  | |  | |  | |
| *Hystrichosphaeridium tubiferum* | 2,5 |  | |  | |  | |  | |  | |  | |
| *Impagidinium dispertitum* | 1 |  | |  | |  | |  | |  | |  | |
| *Lejeunecysta hyalina* | 1 |  | |  | |  | |  | |  | |  | |
| *Lentinia wetzelii* | 7 |  | |  | |  | |  | |  | |  | |
| *Melitasphaeridium pseudorecurvatum* | 5 |  | |  | |  | |  | |  | |  | |
| *Membranosphaera* sp. B in Heilmann-Clausen 85 | 0 |  | |  | |  | |  | |  | |  | |
| *Microdinium* spp. | 1 |  | |  | |  | |  | |  | |  | |
| *Nematosphaeropsis* spp. | 0 |  | |  | |  | |  | |  | |  | |
| *Operculodinium eisenacki* | 20 |  | |  | |  | |  | |  | |  | |
| *Operculodinium microtrianum* | 0 |  | |  | |  | |  | |  | |  | |
| *Operculodinium* spp. | 10 |  | |  | |  | |  | |  | |  | |
| *Palaeocystodinium lidiae* | 0 |  | |  | |  | |  | |  | |  | |
| *Palaeotetradinium minusculum* | 1 |  | |  | |  | |  | |  | |  | |
| *Phthanoperidinium crenulatum* | 8 |  | |  | |  | |  | |  | |  | |
| *Polysphaeridium subtile* | 4 |  | |  | |  | |  | |  | |  | |
| *Senegalinium? dylwenense* | 4 |  | |  | |  | |  | |  | |  | |
| *Spiniferella cornuta* | 0 |  | |  | |  | |  | |  | |  | |
| *Spiniferites* spp. | 158 |  | |  | |  | |  | |  | |  | |
| *Thalassiphora delicata* | 0 |  | |  | |  | |  | |  | |  | |
| *Thalassiphora patula* | 4 |  | |  | |  | |  | |  | |  | |
| *?Thalassiphora?* | 0 |  | |  | |  | |  | |  | |  | |
| broaken chorate dinocyst (gonyaulacoid) | 10 | 1 | |  | |  | |  | |  | |  | |
| *broaken gonyaulacoids* | 1 |  | |  | |  | |  | |  | |  | |
