# Supplementary material for: Correction: First Clarkforkian Equivalent Land Mammal Age in the Latest Paleocene Basal Sparnacian Facies of Europe: Fauna, Flora, Paleoenvironment and (Bio)stratigraphy
Source: PLoS One. 2014 Mar 25;9(3):e93249. doi: 10.1371/journal.pone.0093249 (PMC3965540; doi:10.1371/journal.pone.0093249)
Supplement: File S2 — Republished, corrected article [file pone.0093249.s002.pdf]

OPEN ACCESS Freely available online
PLOS | ONE

First Clarkforkian Equivalent Land Mammal Age in the Latest Paleocene Basal Sparnacian Facies of Europe: Fauna, Flora, Paleoenvironment and (Bio)stratigraphy

Thierry Smith[1]*, Florence Quesnel[2], Gaël De Plöeg[3], Dario De Franceschi[4], Grégoire Métais[4], Eric De Bast[1], Floréal Solé[1], Annelise Folie[1], Anaïs Boura[4], Julien Claude[5], Christian Dupuis[6], Cyril Gagnaison[7], Alina Iakovleva[8], Jeremy Martin[9], François Maubert[10], Judicaël Prieur[3], Emile Roche[11], Jean-Yves Storme[12], Romain Thomas[4], Haiyan Tong[13], Johan Yans[12], Eric Buffetaut[14]

1 Direction Opérationnelle Terre et Histoire de la Vie, Institut royal des Sciences naturelles de Belgique, Bruxelles, Belgium, **2** DGR/GAT, Bureau de Recherches Géologiques et Minières (French Geological Survey) et UMR 7327 CNRS-Université d'Orléans-BRGM, Orléans, France, **3** Centre Permanent d'Initiatives pour l'Environnement des Pays de l'Oise, Verberie, France, **4** Centre de recherche sur la paléobiodiversité et les paléoenvironnements (UMR7207 CNRS-MNHN-UPMC), Muséum National d'Histoire Naturelle et Université Pierre et Marie Curie, Paris, France, **5** Institut des Sciences de l'Évolution de Montpellier, Université de Montpellier 2, Montpellier, France, **6** Géologie GFA, Faculté Polytechnique, Université de Mons, Mons, Belgium, **7** Institut Polytechnique LaSalle-Beauvais, Beauvais, France, **8** Geological Institute, Russian Academy of Sciences, Moscow, Russia, **9** School of Earth Sciences, University of Bristol, Bristol, United Kingdom, **10** Lafarge Granulats Seine Nord, Paris, France, **11** Département de Géologie, Université de Liège, Liège, Belgium, **12** Département de Géologie, Université de Namur, Namur, Belgium, **13** Palaeontological Research and Education Centre, Mahasarakham University, Kantarawichai, Thailand, **14** CNRS (UMR 8538), Laboratoire de Géologie, Ecole Normale Supérieure, Paris, France

Abstract

The Paleocene-Eocene Thermal Maximum (PETM) is correlated with the first occurrences of earliest modern mammals in the Northern Hemisphere. The latest Paleocene Clarkforkian North American Land Mammal Age, that has yielded rodents and carnivorans, is the only exception to this rule. However, until now no pre-PETM localities have yielded modern mammals in Europe or Asia. We report the first Clarkforkian equivalent Land Mammal Age in the latest Paleocene deposits of the basal Sparnacian facies at Rivecourt, in the north-central part of the Paris Basin. The new terrestrial vertebrate and macroflora assemblages are analyzed through a multidisciplinary study including sedimentologic, stratigraphic, isotopic, and palynological aspects in order to reconstruct the paleoenvironment and to evaluate biochronologic and paleogeographic implications. The mammals are moderately diverse and not abundant, contrary to turtles and champsosaurs. The macroflora is exceptional in preservation and diversity with numerous angiosperms represented by flowers, fruits, seeds and wood preserved as lignite material, revealing an abundance of Arecaceae, Betulaceae, Icacinaceae, Menispermaceae, Vitaceae and probably Cornaceae. Results indicate a Late Paleocene age based on carbon isotope data, palynology and vertebrate occurrences such as the choristoderan *Champsosaurus*, the arctocyonid *Arctocyon*, and the plesiadapid *Plesiadapis tricuspidens*. However, several mammal species compare better with the earliest Eocene. Among these, the particular louisinid *Teilhardimys musculus*, also recorded from the latest Paleocene of the Spanish Pyrenees, suggests a younger age than the typical MP6 reference level. Nevertheless, the most important aspect of the Rivecourt fauna is the presence of dental remains of a rodent and a "miacid" carnivoran, attesting to the presence of two modern mammalian orders in the latest Paleocene of Europe. Interestingly, these two groups are also the only modern groups recorded from the latest Paleocene of North America, making Rivecourt the first direct equivalent to the Clarkforkian Land Mammal Age outside of North America.

Citation: Smith T, Quesnel F, De Plöeg G, De Franceschi D, Métais G, et al. (2014) First Clarkforkian Equivalent Land Mammal Age in the Latest Paleocene Basal Sparnacian Facies of Europe: Fauna, Flora, Paleoenvironment and (Bio)stratigraphy. PLoS ONE 9(1): e86229. doi:10.1371/journal.pone.0086229

Editor: Richard J. Butler, University of Birmingham, United Kingdom

Received June 25, 2013; **Accepted** December 7, 2013; **Published** January 29, 2014

Copyright: © 2014 Smith et al. This is an open-access article distributed under the terms of the Creative Commons Attribution License, which permits unrestricted use, distribution, and reproduction in any medium, provided the original author and source are credited.

Funding: This work was supported by projects MO/36/020 and BR/121/A3/PALEURAFRICA of the Federal Science Policy Office of Belgium (to TS; http://www.belspo.be), and Research cooperation contract funded by the BRGM ("Régolithe" and "Paléosurface éocène–PETM" research projects; to FQ, TS, CD, AI, ER and JYS; http://www.brgm.fr). The funders had no role in study design, data collection and analysis, decision to publish, or preparation of the manuscript.

Competing Interests: One of the authors (FM) is employed by the commercial company Lafarge Granulats Seine Nord. This does not alter the authors' adherence to all the PLOS ONE policies on sharing data and materials.

* E-mail: thierry.smith@naturalsciences.be

Introduction

The eastern part of the Paris Basin in northern France is one of the most representative areas for Late Paleocene mammals in Europe. The Cernay-Berru deposits of the Châlons-sur-Vesles Formation have yielded typical index taxa such as the plesiadapiform *Plesiadapis tricuspidens*, the arctocyonid *Arctocyon primaevus* and the "condylarth" *Pleuraspidotherium aumonieri* [1]. The nearby Belgian Basin has yielded the earliest Eocene mammals of Europe in the Dormaal Member of the Tienen Formation, which is correlated with the beginning of the Paleocene-Eocene Thermal Maximum (PETM) and associated with a negative Carbon Isotope Excursion (CIE) [2]. The Dormaal fauna includes the earliest modern mammals of Europe such as the primate *Teilhardina belgica*, the artiodactyl *Diacodexis gigasei* and several species of ischyromyid

rodents, hyaenodontans and "miacid" carnivorans [3]–[5]. Cernay-Berru is reference-level MP6 of the mammalian biochronological scale for the European Paleogene [6]–[7] and is commonly viewed as equivalent to the Late Tiffanian North American Land Mammal Age (NALMA), whereas Dormaal is reference-level MP7 and is correlated with the beginning of the Wasatchian NALMA [2], [8]–[9]. However, up to now, no terrestrial vertebrate assemblage from Europe has been considered coeval with the Clarkforkian NALMA, which is nested between Tiffanian and Wasatchian ages and represents the latest Paleocene in North America.

Here we report the discovery of a new vertebrate assemblage in a basal Sparnacian facies of the Petit Pâtis quarry in the locality of Rivecourt (Oise), in the north-central part of the Paris Basin, along the Oise River between Compiègne and Creil (Fig. 1. and Fig. 2). Among the mammals found at Rivecourt are plesiadapiforms, artocyonids, "condylarths", a rodent and a "miacid" carnivoran. The fauna and flora, both intriguing, indicate significantly different environments than the nearby Houdancourt "Le Quesnoy" site [10]–[14], whose fauna is MP7 in age. The exceptional plant assemblage shows a high Paleocene diversity, especially regarding the reproductive remains preserved as lignitic, carbonized or pyritized organs. The depositional environment of the basal "Sparnacian" lignitic sediments containing the new terrestrial assemblage of Rivecourt can be reconstructed as can the vegetation pattern of the landscapes surrounding the site. The stratigraphic position of Rivecourt is refined in relation to the position of the Paleocene-Eocene boundary, and the paleontological content of the assemblage is integrated into the European mammalian biochronology and compared with the NALMA chronology.

Geological setting

In the area of the Paris Basin of interest to this study (Fig. 1), lignitic sediments are present in two main stratigraphic units, as evidenced in boreholes [15]–[16]. Within the upper part of the Bracheux Formation (= marine Upper Thanetian sands, NP9a, [17]), the sediments indicate fluvial to fluvio-estuarine depositional environments, contain terrestrial plant remains and often some pyrite as well, and were termed "Sables ligniteux supérieurs" (Upper lignitic sands) by previous authors [18]–[19]. Lignite beds and lignitic lacustrine marls and fluvial sands have also been described from the bottom of the classical Sparnacian facies [20]. These "Sparnacian" units are very rich in vertebrate (mammals, crocodiles, turtles) and plant fossils (especially wood fragments, trunks and stumps, occasionally with amber, seeds, and fruits). Pyrite is very abundant and disseminated, forming nodules, cementing sediments, and very often fossils as well.

Both these lignitic units may be present in the vicinity of Rivecourt as shown on the cross section of the Figure 1. Discovery of new outcrops where the exact position of the Paleocene-Eocene boundary can be located is a critical step in revising the complex lithostratigraphic nomenclature of the Paris Basin "Sparnacian" (see [17] for an overview).

Results

Sedimentology and stratigraphy

The composite section of the Petit Pâtis quarry succession at Rivecourt has been reconstituted by careful field observations including tracing contacts of all subsections (red lines on Fig. 2). Although the vertical dimension of the exposure is not of remarkable extent, the horizontal dimension is much better exposed (Fig. S1). Sedimentological and stratigraphic observations reveal a marine unit at the base overlain by fluvial units (Fig. 3 and Additional data in appendix). The marine unit is present throughout the quarry and contains a glauconiferous and bioturbated dark green-grey fine sand, mostly composed of quartz grains (without any carbonate), very well sorted and unimodal.

The fluvial units can be subdivided into 5 sets of sand beds observed in subsections RIVE 2, 3, 5, 6 and 1 (Figs. 2 and 3). These sands are more or less coarse, and are also very rich in quartz grains (without carbonate, but containing some flint grains) and organic matter as well as fossil material. The erosive base surfaces and filling channels of the sands are metric to hectometric in width and decimetric to plurimetric in thickness. The color varies from light grey to black depending on the organic matter content. The sediment composition consists mainly of poorly sorted sands and of lignite, with various proportions of flint gravel and mud. The lateral variation is apparent both in relative abundance of the types of sediment, sedimentary structures, grain size and sorting.

The section alternates horizontal bedding and planar to trough cross bedding,and occasionally exhibits distorted bedding. Some beds contain abundant fossils, including decimetric to metric tree trunk debris or charcoal clasts. The whole section is characterized by variable pyrite concentration, often locally abundant and cementing the sand. The direction of dip of the cross-beds observed in RIVE 2 shifts from 265° to 280°N from Unit I to Unit VI and the angle of dip ranges between 10° and 30°. In RIVE 3 the direction of dip of the cross-beds is very homogeneous (205°N) and the angle of dip ranges between 7° and 16° (Table S1). The succession ends with the RIVE 1 subsection consisting of 1 m of massive to laminated black and pyritic fine lignite, without wood debris or macroflora preserved, but with very thin pale grey fine sand beds (also laminae) that are often discontinuous (lenticular to wavy bedding).

Carbon isotope data

Carbon isotope analysis has been performed on the bulk organic matter from the sediments in order to complement the biostratigraphic data and to help situate the Rivecourt deposits and especially the vertebrate and plant levels with respect to the PETM.

Total organic carbon (TOC) values range from 0.06% to 0.30% in the sandy units, almost 2.0% in the lignitic sand, 6.5% to 19.7% in sands rich in lignitic and pyritized seed, fruit and flower fossils, 1.5% in the laminated clay and 15.5% to 34.7% in the lignitic unit at the top of the succession (Table S2). In the whole Rivecourt succession, the $\delta^{13}C_{org}$ values range from −24.5‰ to −26.6‰ (Fig. 3), and −24.4‰ for an isolated charcoal pebble. The maximal and minimal $\delta^{13}C_{org}$ values were obtained in the very coarse sands with fossils of different subunits, while they are very homogeneous in the fine lignitic unit at the top at around −26‰. No Carbon Isotopic Excursion (CIE) onset similar to the one defined at the Paleocene-Eocene boundary (negative shift of 2.5 to 4‰ or more; [21]) can be seen in the $\delta^{13}C_{org}$ curve.

Palynology

Among the six samples analyzed, the sample RIVE 2-0, collected in the glauconiferous sand of the basal marine Unit 0, is very rich in palynomorphs and contains dinoflagellate cysts (Table S3; Fig. S2). The five other samples collected in the overlying fluvial units are almost devoid of dinocysts (0 to 3 at the bottom, probably reworked from the marine sands) and contain less spores and pollen grains than in the basal glauconiferous sand.

Dinocyst assemblage. Marine palynomorphs of the basal Unit 0 revealed a rich and diverse palynological assemblage

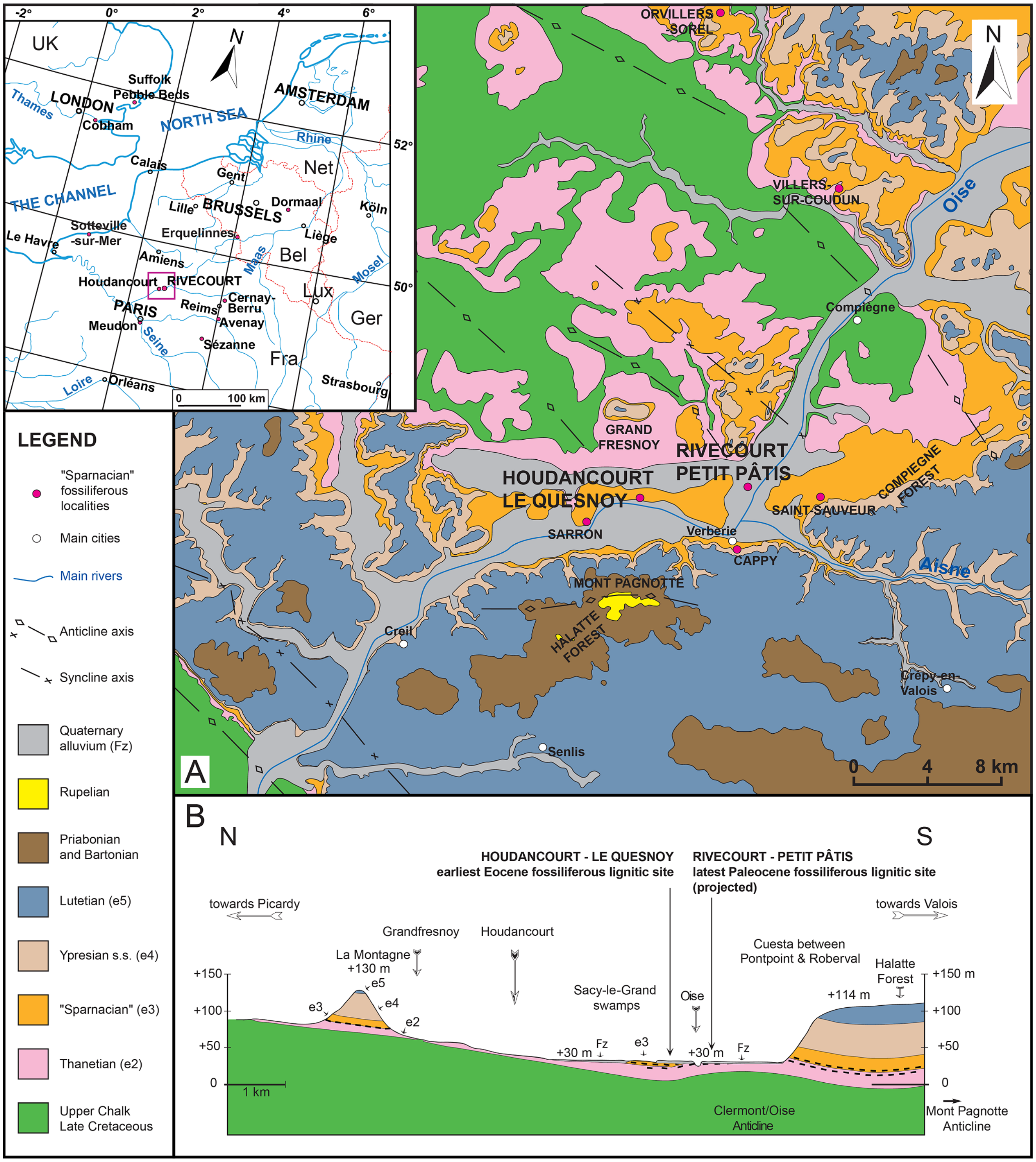

Figure 1. Position of Petit Pâtis Quarry and geological setting around the Rivecourt and Houdancourt fossiliferous sites. (A) Simplified geological map (cf. pink rectangle on the geographical sketch) redrawn from the BRGM geological maps on 1/50,000 scale modified, Compiègne (N°104, [90]), Attichy (N°105, [91]), Villers-Côterêts (N°129, [92]), Senlis (N°128, [93]), Creil (N°127, [94]), Clermont (N° 103, [95]), Saint-Just-en-Chaussée [96], Montdidier (N°81, [97]) and Chauny (N° 82, [98]) showing the stratigraphic relationship and structural setting. (B) Geological cross section, drawn from the BRGM 1/50,000 geological maps of Compiègne (N°104, [90]) to the North and Senlis (N°128, [93]) to the South. Elevation is given in meters above sea level and the thick dotted lines correspond to the lignitic beds of the Upper Thanetian "Sables Ligniteux Supérieurs" and the first "Sparnacian" units.
doi:10.1371/journal.pone.0086229.g001

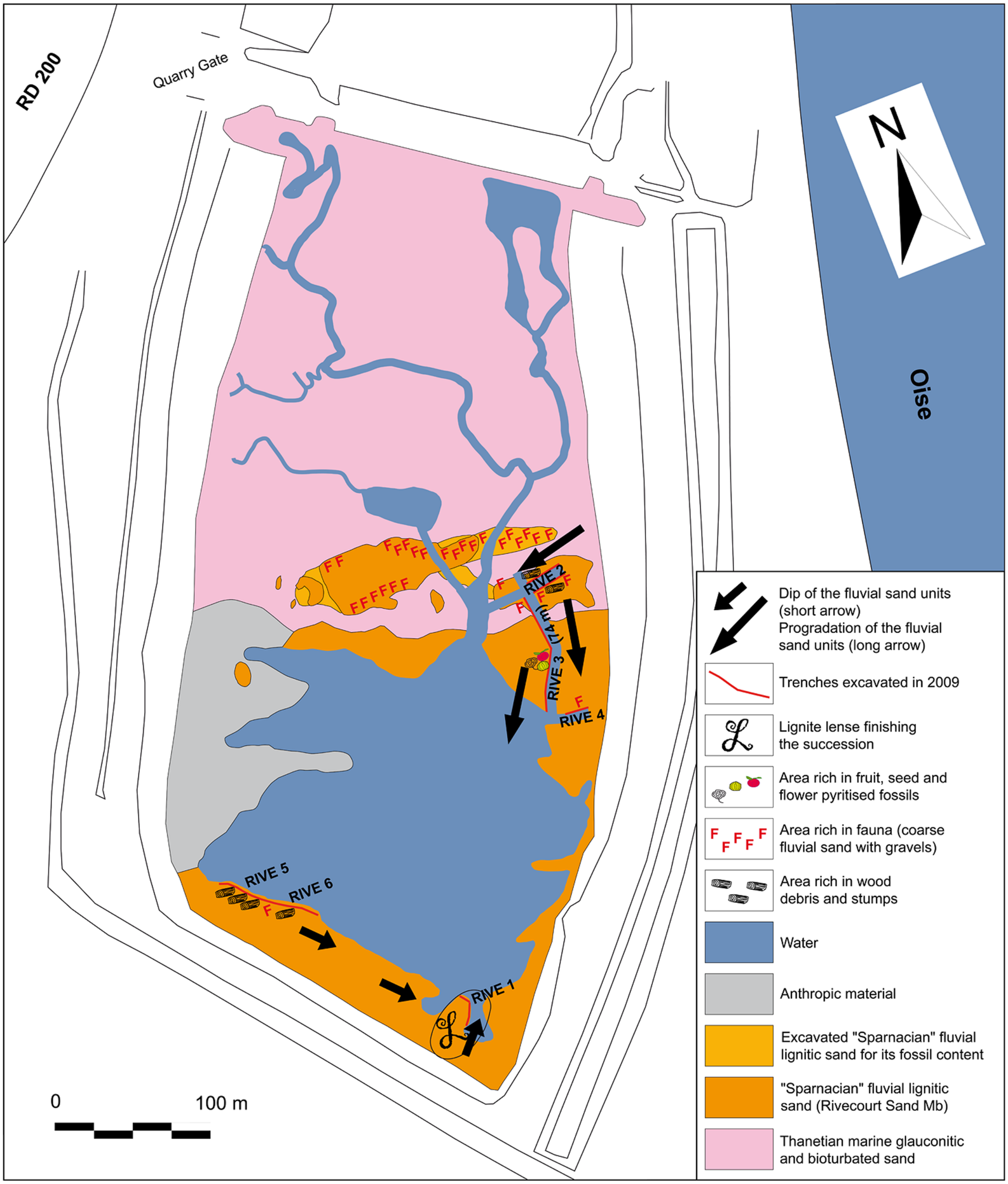

Figure 2. Geological map of the Petit Pâtis Quarry in Rivecourt. Situation below the extracted Oise alluvium during the field work in summer 2009.
doi:10.1371/journal.pone.0086229.g002

dominated by dinoflagellate cysts (87%), with common acritarchs (~11%) and rare prasinophytes. The dinocyst assemblage is clearly dominated by *Spiniferites* spp. (~58%), *Operculodinium* spp. (~12%). *Areoligera coronata* and *Phthanoperidinium crenulatum* represent each only ~3% of the assemblage. The stratigraphically important species are *Alisocysta margarita*, *Deflandrea oebisfeldensis*, *Phthanoperidinium crenulatum*, *Hystrichosphaeridium tubiferum*, *Melitasphaeridium pseudorecurvatum* and *Palaeocystodinium lidiae*.

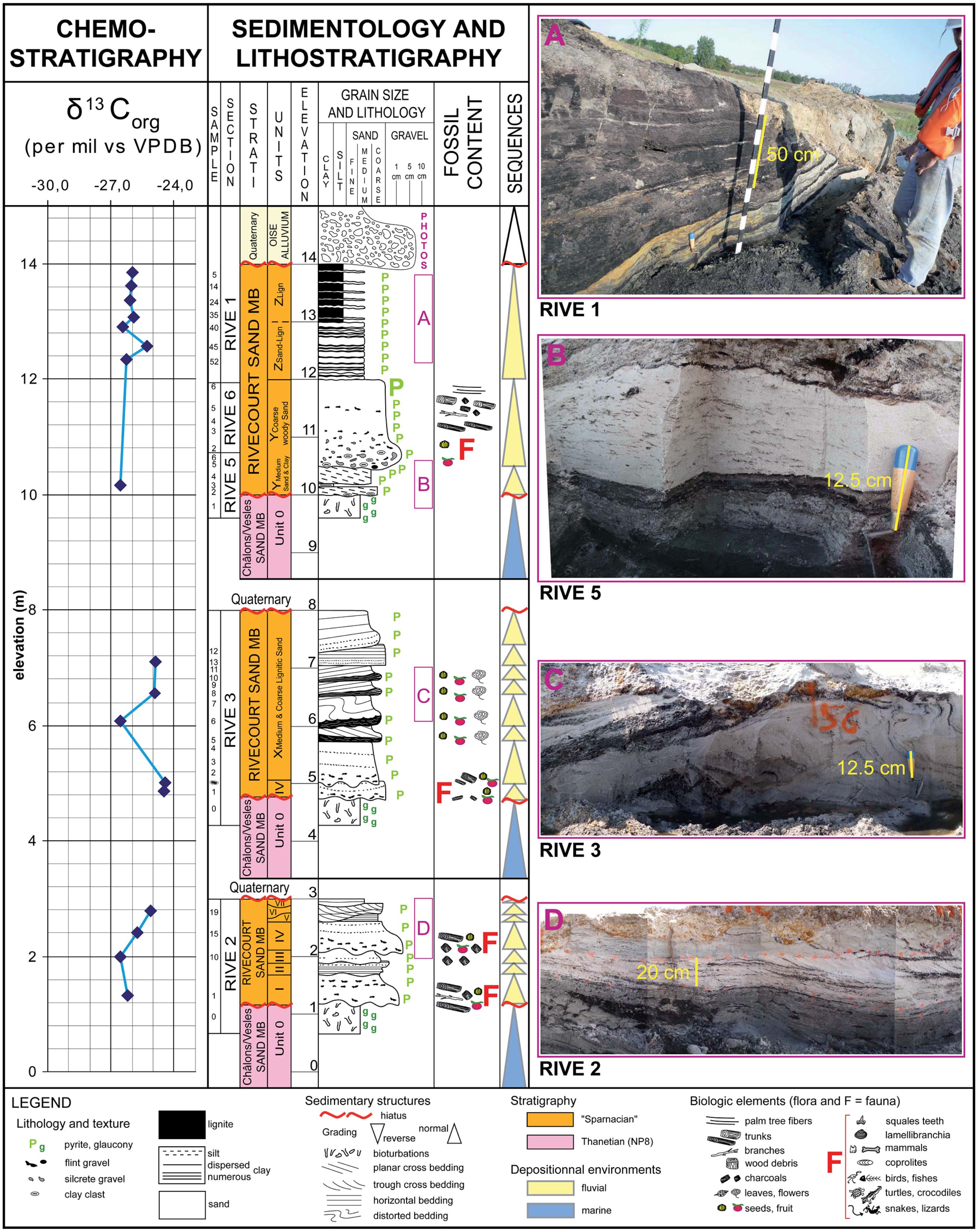

Figure 3. Lithostratigraphy and carbon isotopes on dispersed organic carbon of the Petit Pâtis Quarry in Rivecourt. Sedimentologic log of the composite section, photos of the main facies, stratigraphic data, position of the subsections studied, samples collected, fauna and flora studied, and δ^{13}Corg curve. The person in the picture is one of the authors of the present paper (GDP).
doi:10.1371/journal.pone.0086229.g003

Pollen and spore content. The sporopollinic assemblage is very homogeneous all along the succession, also in the lignitic unit at the top (Additional data in appendix). It is dominated by angiosperms taxa (73 to 88%). The gymnosperm pollen abundance decreases from the marine and first fluvial sand (22 to 24%) to the top of the fluvial units (12 to 9%). The fern spores are not abundant (less than 5%) and the bryophyte spores are rare and only present in the basal marine sand (1%).

The basal marine unit: The angiosperm taxa are dominated by Juglandaceae (*Triatriopollenites platycaryoides* and T. *engelhardtioides*: 16%), *Plicapollis pseudoexcelsus* (13%), *Tricolpopollenites* (12%), *Triatriopollenites rurensis, T. belgicus* and *T. roboratus/aroboratus* (11%) and *Subtriporopollenites* (6%). Gymnosperm taxa are well represented with *Pityosporites* (15%) and *Inaperturopollenites* (8.7%). *Tricolpopollenites henrici/microhenrici* (pre-*Quercus*) pollen grains are also rather significant (6%). *Intratriporopollenites microinstructus*, *Tricolpopollenites hians*, *Triatriopollenites pseudovestibulum*, *Subtriporopollenites magnoporatus magnoporatus* and *tectopsilatus* are present. *Normapolles* taxa represent almost 17% of the pollen and spore content, they are dominated by *P. pseudoexcelsus* (12.5%) but rather diversified with *Pompeckjoidaepollenites subhercynicus*, *Nudopollis terminalis* and *N. endangulatus*, *Basopollis atumescens*, *Sporopollis pseudoporites*, *Interpollis supplingensis* and *Stephanoporopollenites hexaradiatus*. *Monocolpopollenites* pollens are rare (1%); Tetracolporate, *Sparganiaceaepollenites* and *Milfordia* pollens are very rare (0.3%).

The overlying fluvial units: The angiosperm taxa are dominated by *P. pseudoexcelsus* (23 to 37%), Juglandaceae (6 to 19%), *Subtriporopollenites* (8 to 16%), *Triatriopollenites rurensis, T. belgicus* and *T. roboratus/aroboratus* (6 to 11%). Gymnosperm taxa are less abundant than in basal marine unit with *Pityosporites* (8%) and *Inaperturopollenites* (4%). *Intratripollenites* (Lime) and *T. henrici/microhenrici* (pre-Quercus) pollen grains are present but rare (1 to 2%). *Triporopollenites robustus*, *Subtriporopollenites constans*, *S. anulatus*, *S. magnoporatus magnoporatus* and *tectopsilatus*, *S. spissoexinus* and *Intratriporopollenites microinstructus* are present. Other *Normapolles* taxa than *P. pseudoexcelsus* are not very abundant (4%) but rather diversified with *Pompeckjoidaepollenites subhercynicus*, *Nudopollis terminalis*, *N. endangulatus*, *Basopollis atumescens*, *Sporopollis pseudoporites*, *Interpollis supplingensis*.

Tetracolporate pollen grains are very rare (less than 1%) and only present in the first fluvial sand. *Monocolpopollenites* pollens are rare (1%); Sparganiaceae and *Milfordia* pollen grains are present but also rare (1 and 1.5% respectively).

Macroflora

Some beds, such as those of Units I and IV of RIVE 2 subsection and Unit X of RIVE 3 subsection, are especially rich in fossil wood, seeds and fruits (Fig. 4). The macro plant remains are nevertheless spread throughout the site in lignite lenses or dispersed in the sandy layers. The remains mainly consist of lignified and/or sclerified organs, such as stems, roots, rachis of leaves, seed fruits, but also flowers and inflorescences. The plant fragments are preserved as lignitic (all plant organs), carbonized (mainly wood fragments) or pyritized (fruits and seeds p.p.) material.

Vegetative structures. No leaf lamina, nor fossil resin were found in the lignite layers of this site.

The wood samples, especially the carbonized pieces, are slightly blunted, suggesting a short distance transport. The wood fragments consist of about 80% of angiosperms and 20% of conifers. The structures of some show more or less distinct growth ring limits.

Reproductive organs. The collected reproductive material represents several thousand fruits and seeds, whose study is in progress. The size of these elements ranges between 1 and 20 mm. Bigger sized fruits are rare. Preliminary results demonstrate the presence of numerous angiosperm families (Table 1): Anacardiaceae, Arecaceae, Betulaceae, ?Cornaceae, Icacinaceae, Menispermaceae, Piperaceae, and Vitaceae are all relatively abundant. Among them, Icacinaceae and Menispermaceae endocarps, Vitaceae seeds and Betulaceae fruits show the highest frequency. The well-preserved, lignitized specimens provide both anatomical and morphological data potentially exploitable with the help of microtomography or thin sections. Icacinaceae are mainly represented by diverse species of *Iodes* Blume, close to *I. multireticulata* Reid & Chandler 1933 from the London Clay Formation, but they show differences partially due to taphonomy and different stages of preservation. They probably correspond to different closely related biological taxa. The same is observed on the diverse Menispermaceae of Oise (*Wardensheppeya* Eyde 1970). Vitaceae are common, with a few species of *Ampelocissus* Planch. and *Vitis* L. The relationships between these fossils and extant or extinct (London Clay, England; Dormaal, Belgium; Clarno Formation, Oregon, USA) taxa are still not well understood. Nevertheless, the affinities of the taxa of this assemblage provide further evidence of megathermal vegetation.

Flower remains are less numerous, mainly preserved as lignite and are slightly compressed, highlighting the constraints of the softness of the material during the first steps of fossilisation. Triflowered cymose inflorescences close in organisation to modern *Fagales* are abundant. Other types of small flowers are also present. They are mainly of pentamerous types but still unidentified.

Mammals

By comparison with other Paleogene localities of the Paris Basin, mammal remains such as teeth and bones are relatively rare at Petit Pâtis quarry. They are concentrated in the lower part of channels, mostly near the contact with the underlying marine unit. A total of 230 mammal dental remains have been collected, among which about 180 are diagnostic. Diversity of the mammals is calculated based on 128 jugal teeth, P4 to M3 and p4 to m3 (Fig. 5) and have permitted identification of the following groups (Table 2).

Plesiadapiforms. This group is represented by at least 3 taxa. The most abundant mammal species is a relatively large plesiadapid (Fig. 6). The size and morphology of the incisors, and upper and lower molars from Rivecourt match perfectly those of *Plesiadapis tricuspidens* Gervais, 1877 from Cernay and Berru (Table 3). The size of the m3s of *Plesiadapis tricuspidens* from Rivecourt is slightly smaller than that of specimens from Berru, but similar to specimens from Cernay. The length of the I1 from Rivecourt attributed to *P. tricuspidens* is shorter than the average in Berru, but still falls within the documented variability, even though molars from Berru are known to be larger than in Cernay. The I1/m3 length ratio of specimens from Rivecourt (0.92) is similar to specimens from Cernay and Berru (0.86).

Mid-sized species of plesiadapiforms are represented only by a few teeth. An upper incisor shares with plesiadapids the tricuspid morphology, with the anterocone being the largest cusp and a relatively symmetrical apex of the tooth. As for I1s of closely related plesiadapoid carpolestids, they are characterized by the absence of mediocone. The specimen from Rivecourt differs from those of all species of *Plesiadapis* by the similarly sized mediocone and laterocone, and by several small cusps occupying a wide posterocone region instead of one single large posterocone. The species *Chiromyoides campanicus* has the most similar upper incisor morphology based on the illustrations from Russell [1] and Gingerich [22] with the exception of the larger size than that of the

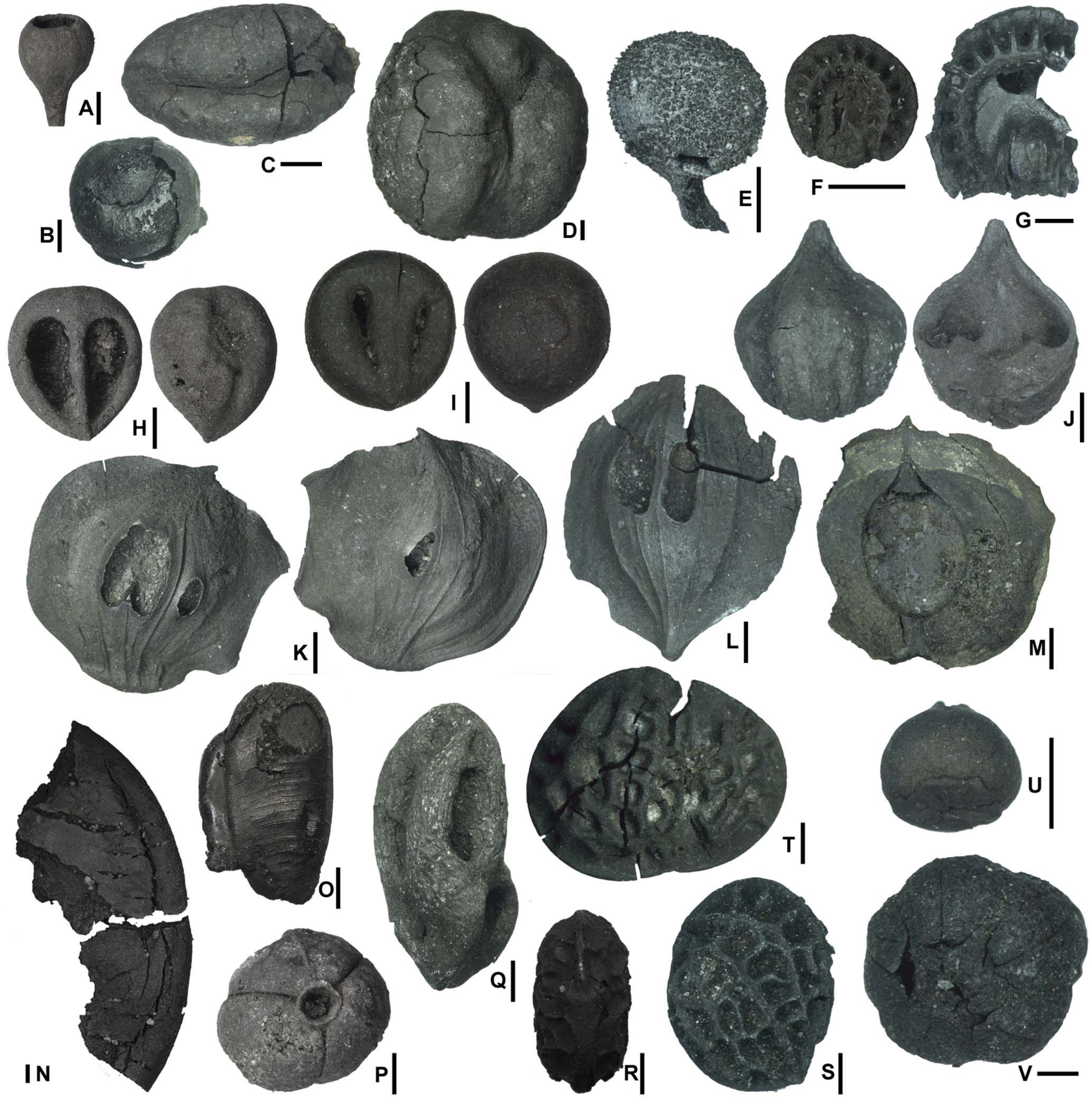

Figure 4. Macroflora from the latest Paleocene of the Petit Pâtis quarry in Rivecourt. (A) Lauraceae, cupule RIV.PPB 1, *Litsea pyriformis* Reid & Chandler; (B–D) Arecaceae (B) RIV.PPB 2, *Palmospermum excavatum* R. & C.; (C) RIV.PPB 3, *Phoenix* sp.; (D) RIV.PPB 4, *Oncosperma anglica* R. & C.; (E) Schisandraceae, fruit RIV.PPB 5, *Schisandra* sp; (F–G) Menispermaceae, endocarps (F) RIV.PPB 6, *Wardensheppeya davisii* (Chandler) Eyde, (G) RIV.PPB 7, *Stephania* sp.; (H–I) Vitaceae, seeds (H) RIV.PPB 8, *Ampelocissus* sp., (I) RIV.PPB 9, *Vitis* sp.; (J) RIV.PPB 10, *Juglandicarya* R. & C.; (K–M) Betulaceae, fruits cf. *Palaeocarpinus* sp.; (K) RIV.PPB 11, view of the two sides of the same fruit, (L) RIV.PPB 12, a different fruit showing a thinner base; (M) RIV.PPB 13, inner part of an open fruit; (N) ?Fabaceae RIV.PPB 14, seed fragment; (O) Fabaceae RIV.PPB 15, seed with attached arillode; (P) Euphorbiaceae, fruit RIV.PPB 16, *Euphorbiotheca* sp.; (Q) Anacadiaceae, endocarp RIV.PPB 17, aff. *Lannea* sp.; (R–T) Icacinaceae, endocarps (R–S) RIV.PPB 18, *Iodes* cf. *multireticulata* R. & C. in lateral and base views; (T) RIV.PPB 19, *Iodes* sp. 1; (U) ?Lamideae, fruit RIV.PPB 20, *Carpolites* sp. 1; (V) ?Cornaceae/ ?Mastixiaceae, fruit RIV.PPB 21, *Carpolites* sp.2. All figures, scale bars = 1 mm.
doi:10.1371/journal.pone.0086229.g004

incisor from Rivecourt. Moreover, incisors of both taxa present a relatively short and wide crown in comparison with *Plesiadapis*. Additionally the presence of the genus *Chiromyoides* is documented by a fragment of dentary with m3 that displays an antero-posteriorly constricted trigonid with a straight anterior outline and reduced cusps, differing from all species of *Plesiadapis* that display a less constricted trigonid with rounded anterior outline. The m3 of *C. campanicus* is similar in size but differs by a more classical tri-cuspid trigonid. The species from Petit Pâtis could thus represent a new species of *Chiromyoides*. The size of the m3 of *Chiromyoides* sp.

Table 1. Preliminary list of plants (macro-flora) in the Rivecourt assemblage.

CONIFEROPHYTES
Coniferales
Pinaceae
Taxodioxylon sp.
ANGIOSPERMS
Magnolideae
Piperales
Piperaceae
Infrutescence indet.
Laurales
Lauraceae
Litsea pyriformis Reid & Chandler
Monocotyledons
Commelinideae
Arecales
Arecaceae
Palmospermum excavatum Reid & Chandler
Oncosperma anglica Reid & Chandler
Phoenix sp. (seed)
Sabal sp. (petiole fragment)
Eudicotyledons
Ranunculales
Schisandraceae
Seeds indet cf *Schisandra*
Menispermaceae
Wardensheppeya davisii (Chandler) Eyde
Stephania sp.
endocarp indet.
Vitales
Vitaceae
Ampelocissus sp.
Vitis sp.
Rosideae
Fabideae
Fagales
Juglandaceae
Juglandicarya R. & C.
Betulaceae
cf. *Paleocarpinus* (fruits)
tri-flowered cymes indet.
male strobiles indet.
Fabales
Fabaceae
seed indet.
Malvideae
Malvales
Tiliceae
fruits indet.
Malpighiales
Euphorbiaceae
Euphorbiotheca sp.
Sapindales
Anacardiaceae
Lannea sp. (seed)
Asterideae
Cornales
Cornaceae (fruits) indet.
Lamiideae
Icacinaceae
Iodes cf. *multireticulata*
Iodes sp.1
Iodes sp.2

doi:10.1371/journal.pone.0086229.t001

from Rivecourt is slightly smaller (10% difference) than that of *C. campanicus* from Cernay and Berru (Table 3). The I1 from Rivecourt is significantly smaller (20% difference) than that of *C. campanicus*. The I1/m3 length ratio is therefore lower for the specimens from Rivecourt (1.06 vs. 1.24).

The crown of an upper incisor of a very large plesiadapid (about 60% larger than *P. tricuspidens*) displays a particular morphology with a long and strong anterocone and very small mediocone and laterocone. This impressive I1clearly indicates the presence of the genus *Platychoerops* at Petit Pâtis. The length of the I1 of *Platychoerops* sp. from Rivecourt is similar to that of *Plesiadapis cookei* from the latest Paleocene of Wyoming and *Platychoerops daubrei* from the Early Eocene of Mutigny, and slightly larger than that of *Platychoerops russelli* from the Early Eocene of Meudon (Table 3).

Procreodi. Two lower molars of a large arctocyonid are referred to *Arctocyon primaevus* based on morphological features, but the size of the Rivecourt species is somewhat smaller. A full comparison including size variability in this species would require more complete material.

One upper molar of a small arctocyonid is attributed to the genus *Landenodon*. It differs from *L. lavocati* from Cernay by its larger size and tri-lobed outline in occlusal view. It is similar in size and morphology to *L. woutersi* from Dormaal [23]. However, it is possible that this upper molar belongs to *L. phelizoni* from Berru, a taxon only known by a lower jaw that is the same size as *L. woutersi* [24].

Pleuraspidotheriidae. The second-most abundant taxon in Rivecourt is a new Pleuraspidotheriidae. The new genus is much smaller than all other members of the family. The upper molars are relatively similar to those of *Pleuraspidotherium aumonieri* from Cernay and Berru, but much smaller (45% the size of *P. aumonieri*), and show more developed crests, especially on the protocone and pseudohypocone, and no anterior cingulum on M1 and M2. Upper molars of the new taxon are closer in size to *Orthaspidotherium edwardsi* from Cernay and Berru (70% the size of *O. edwardsi*) and share with this species the asymmetrical implantation of the molars in the maxillary, which is not visible in *P. aumonieri* [25]. However, the crests are much less developed in *O. edwardsi* than in the new taxon, and the pseudohypocone is less developed. Lower molars differ from *O. edwardsi* and *P. aumonieri* by their smaller size, more developed crests, more oblique crista obliqua that almost reaches the lingual edge of the molars, more lingually displaced hypoconulid on m1 and m2 resulting in a postcristid that is longer and more

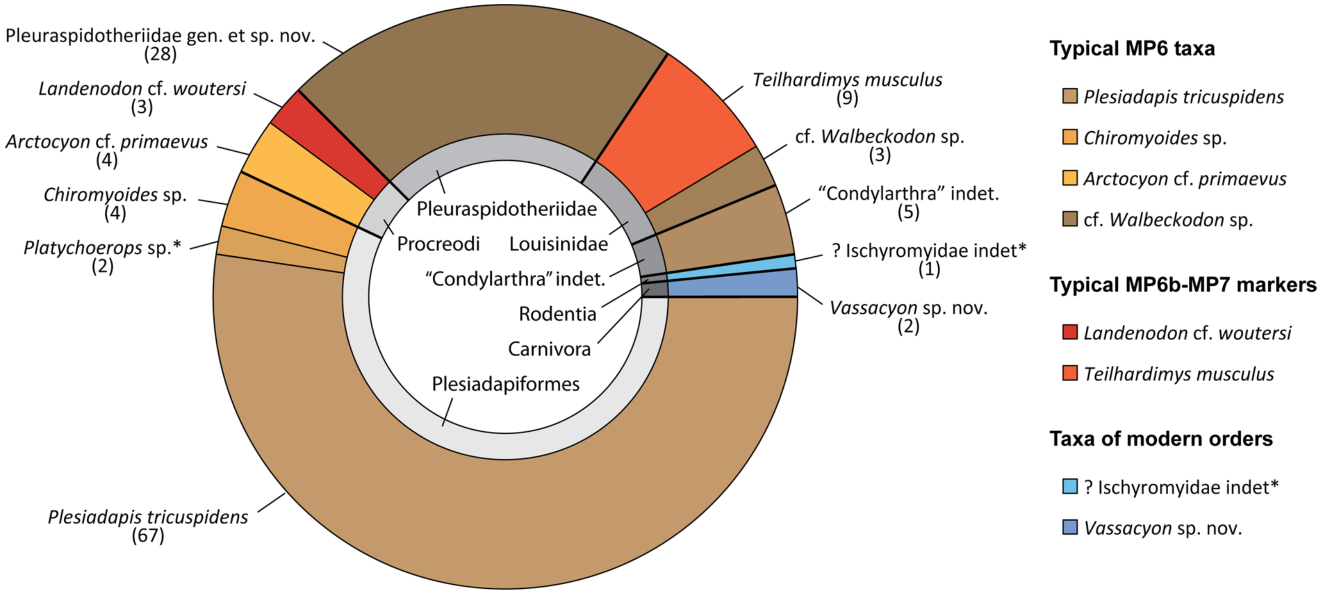

Figure 5. Diversity of the mammal groups present in Rivecourt. Proportions are calculated on the basis of the absolute number of P4 to M3 and p4 to m3 of each taxon with the exception of the rodent and largest plesiadapid *Platychoerops* sp. (* a single incisor for each).
doi:10.1371/journal.pone.0086229.g005

perpendicular to the anteroposterior axis, and the very large hypoconulid lobe on m3. The taxon from Rivecourt differs from the pleuraspidotheriid *Hilalia* and especially from *Parabunodon* respectively from the Early and Middle Eocene of Turkey by a much less transverse development of upper molars [26]. Moreover, it differs from *Parabunodon* by much more developed crests and by the absence of an ectocingulum and by a less reduced parastylar lobe.

Louisinidae. Two fragmentary dentaries belonging to a small louisinid "condylarth" were found. This taxon represents the smallest mammal known from the locality and is similar to *Walbeckodon girardi* in retaining a small paraconid on m2 and in having a proportionally larger m3 with an unreduced hypoconulid [27]. However, it differs from *W. girardi* by its larger size (a little over 10% larger).

The third-most abundant taxon in Rivecourt is another louisinid "condylarth," *Teilhardimys musculus*, represented by all upper and lower jugal teeth including a blade-like p4 [28]. This species is known from the earliest Eocene of Dormaal in Belgium and the latest Paleocene of Tremp in Spain.

Rodents. A long and curved incisor displaying a morphology similar to that of early rodents was found at Rivecourt. This incisor is distinctive from that of multituberculates, which are superficially similar, by the shape of the tooth section, the curvature of the crown, the presence of a thin band of enamel only in the front part of the tooth and the extent of this enamel band towards the base of the tooth.

Carnivorans. Perhaps the most important specimens found in Rivecourt are two trigonids and one dentary of a primitive carnivoran with p4, m1 and m2. The mandible found in Rivecourt (Fig. 6) displays three molars, decreasing in length posteriorly. The m1, which displays a secant morphology, is characterized by a weakly anteriorly projected paraconid (compared to other "miacids"), a basined talonid bearing a developed entoconid, and the presence of a complete labial cingulid. These features are similar to the genus *Vassacyon*, which is known in the Early Eocene of North America, rather than the sole Paleocene "miacid" genus *Uintacyon* [29]. *Vassacyon* was previously unknown in Europe. Moreover, because of its very small size, the specimen from Rivecourt could represent a new species. This record constitutes the first unambiguous occurrence of modern mammals in the European Paleocene.

Other vertebrates

Fishes. Elasmobranch teeth are the most common vertebrate remains found in Rivecourt. Like all other vertebrate remains, they are present only in the lower part of the fluvial units. Thirteen species of sharks and rays have been preliminarily mentioned [30]. In addition to those, we can add three other, rarer taxa: *Hypolophodon sylvestris*, *Coupatezia* sp., and *Paraorthacodus* sp. Curiously, chimeriforms such as cf. *Edaphodon* sp. are not particularly rare. Among marine osteichthyans, dental remains of *Diaphyodus* sp. are abundant. Among freshwater fishes, the presence of *Lepisosteus*, that was only based on a single typical scale found during earlier excavations, was not confirmed by later work. The likely absence of *Lepisosteus* in Rivecourt contrasts totally with the earliest Eocene localities of Dormaal and Le Quesnoy where *Lepisosteus* scales are extremely abundant.

Amphibians. Only caudates are represented in Petit Pâtis (Fig. 7). The amphicoelous isolated vertebrae identified at Rivecourt are attributed to the ambystomatoid Dicamptodontidae based on the vertebral proportions and the lack of interavertebral spinal nerve [31]. They moreover resemble the vertebrae of the genus *Wolterstorffiella* from the Paleocene of Walbeck (Germany; [32]) by being relatively large and wide, and by the articulation surface with the processes being close to the midpoint of the centrum rather than at its posterior end. The Rivecourt vertebrae have nevertheless a lower neural spine than that of *Wolterstorffiella*.

Squamates. Squamates are represented by an amphisbaenian lizard with a robust dentary that does not form a strong angle at the posterior end of the intermandibular symphysis and bears 11 bulbous teeth, indicating that it belongs to the primitive lineage of Paleocene amphisbenians and more precisely to the species *Camptognathosaurus parisiensis* [33]. A large and robust anguimorph is also present at Rivecourt, represented by vertebrae, oval osteoderms and a dentary. Oval osteoderms are also present among necrosaurid taxa [34]. However, dentaries of necrosaurids

Table 2. Preliminary list of vertebrates in the Rivecourt assemblage.

CHONDRICHTHYES
at least 16 species (reworked, see Gagnaison et al 2009)
OSTEICHTHYES
About 10 species (partially reworked, see Gagnaison et al 2009)
AMPHIBIA
Caudata
Scapherpetontidae indet.
REPTILIA
Lacertilia
Anguimorpha indet.
Scincomorpha indet.
Amphisbaenia
Camptognathosaurus parisiensis
Serpentes
Boidae indet.
Crocodylia
Diplocynodontidae indet.
cf. *'Crocodylus' depressifrons*
Choristodera
Champsosaurus sp.
Testudines
Trionychidae
Palaeotrionyx sp.
Trionychidae indet.
Pleurosternidae
Berruchelus sp.
Macrobaenidae indet.
Mongolochelidae indet.
Cheloniidae indet. (reworked)
AVES
Palaeognathae
?Remiornithidae
cf. *Remiornis* sp.
Neognathae
Gastornithidae
cf. *Gastornis* sp.
Neognathae indet.
MAMMALIA
Plesiadapiformes
Plesiadapidae
Plesiadapis tricuspidens
Chiromyoides sp.
Platychoerops sp.
Procreodi
Arctocyonidae
Arctocyon cf. *primaevus*
Landenodon cf. *woutersi*
"Condylarthra"
Pleuraspidotheriidae gen. et sp. nov.
Louisinidae

Table 2. Cont.

Teilhardimys musculus
cf. *Walbeckodon* sp.
"Condylarthra" family indet.
Rodentia
?Ischyromyidae indet.
Carnivora
"Miacidae"
Vassacyon sp. nov.

doi:10.1371/journal.pone.0086229.t002

present more widely spaced teeth than those of Rivecourt. Maxillaries and dentaries also attest the presence of a scincomorph lizard. Teeth apices are bicuspid, and the lingual cusp is reduced compared to the labial cusp [34]–[35]. Finally, snakes are also present at Rivecourt, represented by two short incomplete trunk vertebrae bearing no parazygantral nor paracotylar foramina but presenting small prezygapophyseal processes and paradiapophyses that are well subdivided into parapophyses and diapophyses. These vertebrae are attributed to an undetermined boid [36].

Choristoderans. A very abundant taxon similar to the typically Paleocene long snouted choristoderan *Champsosaurus dolloi* is identified based on dentary fragments and tens of vertebrae [37].

Crocodilians. Two taxa can be recognized. A middle sized animal is identified based on an edentulous dentary fragment with two confluent alveoli for accommodating the double caniniform dentition (3rd and 4th dentary teeth) and is reminiscent of an alligatoroid diplocynodontid (e.g. [38]). Although poorly preserved, the splenial suture does not seem to reach the symphysis and may justify this assignment. A large crocodyloid dentary fragment is similar to "*Crocodylus*" *depressifrons* in having one large caniniform alveolus [39]. Both taxa are also represented by osteoderms, respectively square and more rounded in outline, those attributable to the alligatoroid presenting comparatively larger cupules than in the crocodyloid osteoderms.

Testudines. Turtles are well diversified, represented by scattered isolated elements. A preliminary list can be provided based on the diversity of shell elements and ornamentation. Two trionychid species are present. A species of large size displays an irregular ornamentation consisting of ridges and large pits, and costal plates with rib ends extending laterally. It may correspond to the genus *Palaeotrionyx* (this genus, however, certainly needs to be revised in Europe: see [40], [41]). A second species displays a more regular ornamentation on neural bones consisting of smaller pits. Other specimens, smaller in size and showing a particular ornamentation with numerous ridges on the thin costal and neural plates and low, often fused tubercules on plastral plates, are attributed to the paracryptodiran pleurosternid turtle *Berruchelus russelli* Perez-Garcia, 2012 known from the Late Paleocene of Berru [42]. A fourth taxon of large size, presenting a smooth outer surface, thick peripheral plates with a guttered lateral border, and a few deep pockets on the medial margin into which the extremities of peg-like thoracic rib ends inserted, can be attributed to Macrobaenidae. Within this family, it resembles the large genus *Anatolemys* Khozatsky and Nessov 1979 [43] but displays some differences, such as its narrower cervical scute. A fifth taxon with thick peripheral and nuchal plates displays irregular scute dermo-sulci: coarse growth annuli on peripherals are reminiscent of the morphology of some basal taxa (Paracryptodira, Kallokibotiidae or Mongolochelyidae). A sixth taxon is represented by wide nuchal

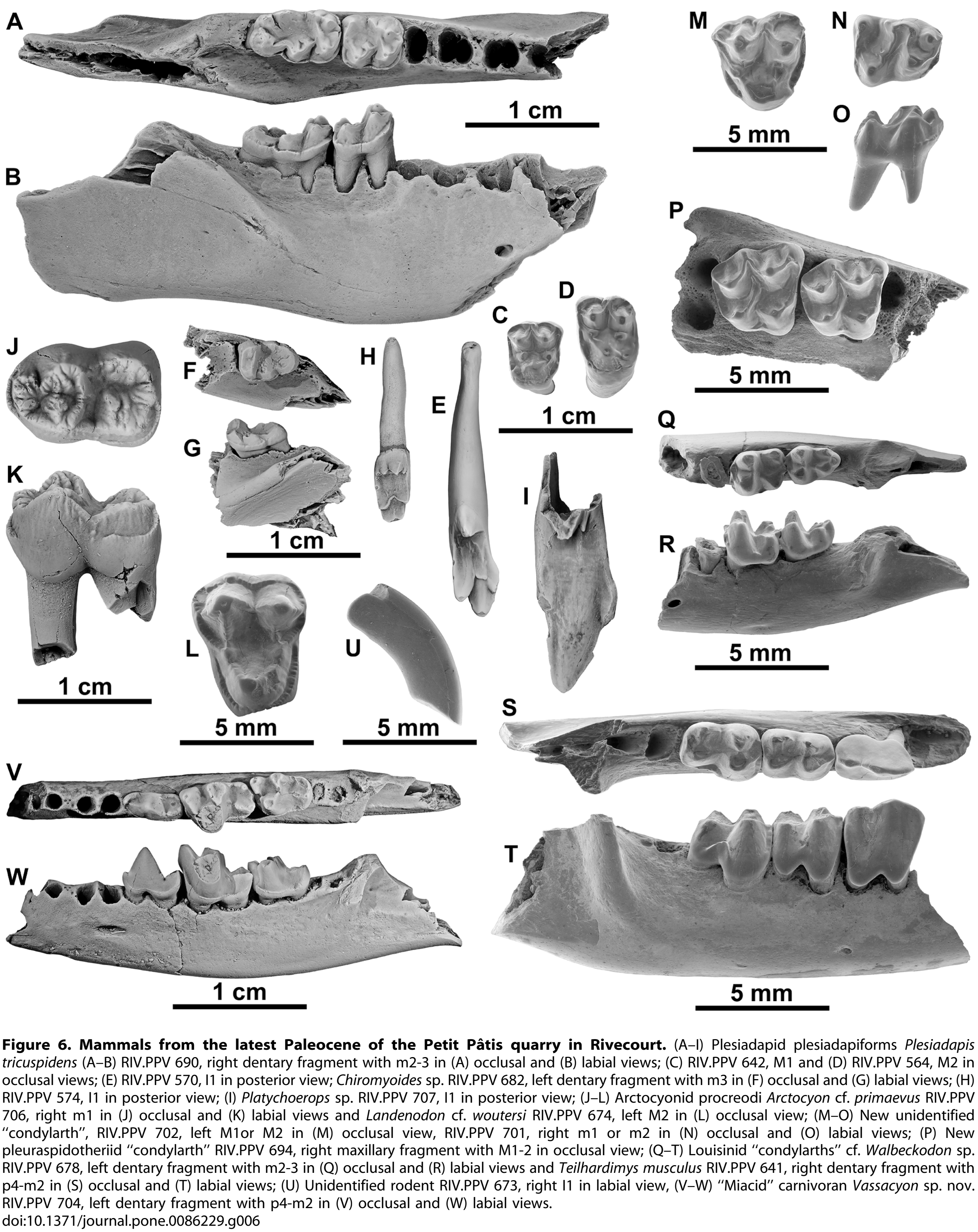

Figure 6. Mammals from the latest Paleocene of the Petit Pâtis quarry in Rivecourt. (A–I) Plesiadapid plesiadapiforms *Plesiadapis tricuspidens* (A–B) RIV.PPV 690, right dentary fragment with m2-3 in (A) occlusal and (B) labial views; (C) RIV.PPV 642, M1 and (D) RIV.PPV 564, M2 in occlusal views; (E) RIV.PPV 570, I1 in posterior view; *Chiromyoides* sp. RIV.PPV 682, left dentary fragment with m3 in (F) occlusal and (G) labial views; (H) RIV.PPV 574, I1 in posterior view; (I) *Platychoerops* sp. RIV.PPV 707, I1 in posterior view; (J–L) Arctocyonid procreodi *Arctocyon* cf. *primaevus* RIV.PPV 706, right m1 in (J) occlusal and (K) labial views and *Landenodon* cf. *woutersi* RIV.PPV 674, left M2 in (L) occlusal view; (M–O) New unidentified ''condylarth'', RIV.PPV 702, left M1or M2 in (M) occlusal view, RIV.PPV 701, right m1 or m2 in (N) occlusal and (O) labial views; (P) New pleuraspidotheriid ''condylarth'' RIV.PPV 694, right maxillary fragment with M1-2 in occlusal view; (Q–T) Louisinid ''condylarths'' cf. *Walbeckodon* sp. RIV.PPV 678, left dentary fragment with m2-3 in (Q) occlusal and (R) labial views and *Teilhardimys musculus* RIV.PPV 641, right dentary fragment with p4-m2 in (S) occlusal and (T) labial views; (U) Unidentified rodent RIV.PPV 673, right I1 in labial view, (V–W) ''Miacid'' carnivoran *Vassacyon* sp. nov. RIV.PPV 704, left dentary fragment with p4-m2 in (V) occlusal and (W) labial views.
doi:10.1371/journal.pone.0086229.g006

and neural plates presenting the morphology of a possibly cheloniid sea turtle. Except for the latter that is probably reworked, all Rivecourt turtles lived in fresh water or were possibly terrestrial (the fifth taxon, for instance). While the trionychids and the cheloniid cannot provide clear stratigraphic indications, the other taxa have never been reported from Early

Table 3. Size and variability of I1 and m3 in different key plesiadapid species.

species	specimen number	mean length	VL	s	mean width	VL	s	authors
**m3**								
Plesiadapis tricuspidens (Berru)	average (63)	7.63	6,8–8,7	0.41	4.46	4,0–5,0	0.25	Gingerich 1976
Plesiadapis tricuspidens (Cernay-Berru)	average (178)	6.65	4,8–8,5	0.80	4.05	2,8–5,0	0.43	Russell 1964
Plesiadapis tricuspidens (Berru)	average (7)	7.56	7,10–7,95	0.25	4.36	3,94–4,61	0.23	coll. RBINS
***Plesiadapis tricuspidens* (Rivecourt)**	average (8)	6.88	6,53–7,27	0.28	3.95	3,81–4,28	0.17	
Chiromyoides campanicus (Cernay)	MNHN CL118	4.58			2.78			cast RBINS
Chiromyoides campanicus (Cernay-Berru)	average (3)	4.53	4,2–4,8	0.25	2.87	2,8–3,0	0.09	Gingerich 1976
***Chiromyoides* sp. (Rivecourt)**	average (3)	4.02	3,75–4,29	0.27	2.68	2,50–2,82	0.14	
**I1**								
**species**	**specimen number**	**mean length**	**VL**	**s**	**width**	**VL**	**s**	**authors**
Plesiadapis tricuspidens (Berru)	Louis coll.	8.1			4.4			Gingerich 1976
Plesiadapis tricuspidens (Berru)	average (5)	6.59	5,93–7,30	0.50	-	-	-	Boyer et al. 2010
***Plesiadapis tricuspidens* (Rivecourt)**	RIV PPV570	6.33			3.51			
Plesiadapis cookei (Clark's Fork Basin)	average (4)	11.28	9,83–12,90	1.12	-	-	-	Boyer et al. 2010
Chiromyoides campanicus	-	5.2			3.6			Gingerich 1976
Chiromyoides campanicus (Cernay)	MNHN CR357	5.9			3.9			Russell 1964
***Chiromyoides* sp. (Rivecourt)**	RIV PPV572	4.25			2.76			
Platychoerops russelli (Meudon)	type specimen	8,3*			4.37			cast RBINS
Platychoerops daubrei (Mutigny)	average (7)	9.52	7,96–11,30	1.07	-	-	-	Boyer et al. 2010
***Platychoerops sp.* (Rivecourt)**	RIV PPV707	10,8*			5.95			

VL: Variation limits, s: standard deviation.
doi:10.1371/journal.pone.0086229.t003

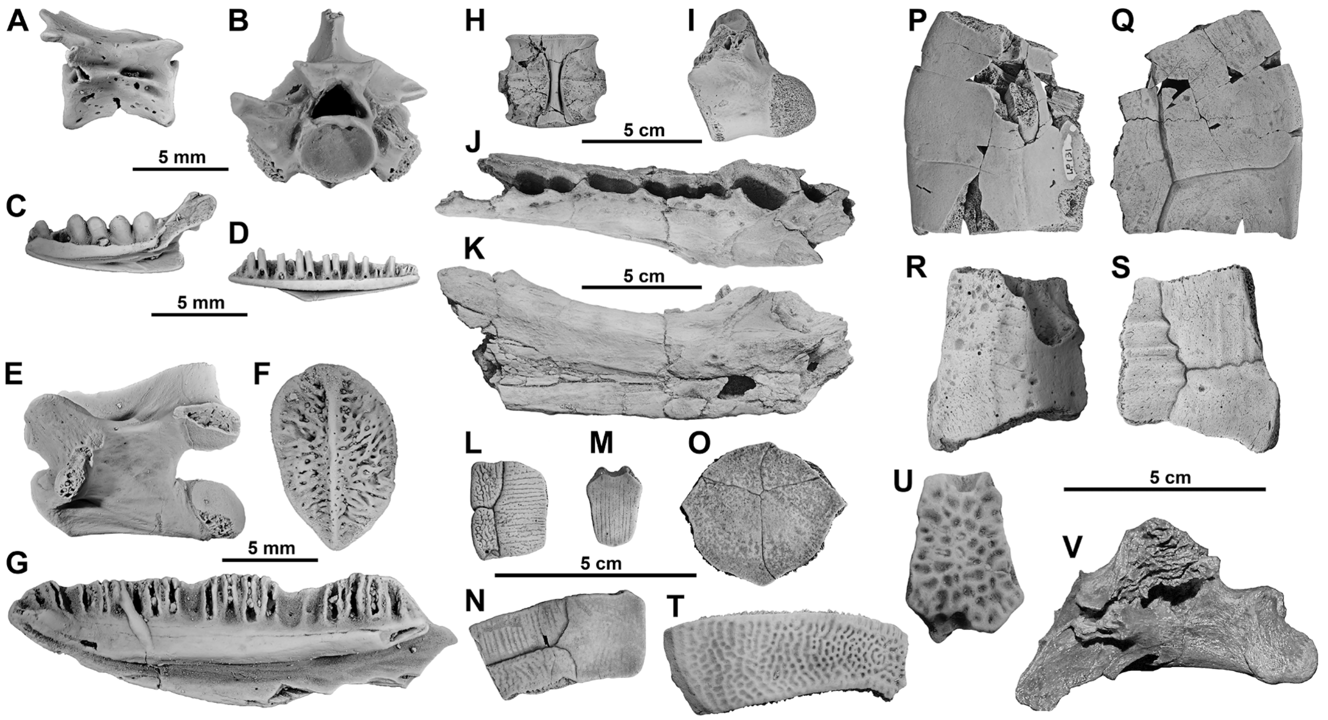

Figure 7. Other vertebrates from the latest Paleocene of the Petit Pâtis quarry in Rivecourt. (A) Ambystomatoid caudate RIV.PPV 397, vertebra; (B) Boid snake RIV.PPV 478, vertebra; (C) Amphisbaenid lizard *Camptognathosaurus parisiensis* RIV.PPV 412, dentary; (D) Scincomorph lizard RIV.PPV 416, dentary, (E–G) Anguid lizard (E) RIV.PPV 441, vertebra; (F) RIV.PPV 423, osteoderm and (G) RIV.PPV 417, dentary; (H) Choristoderan *Champsosaurus* sp. RIV.PPV 708, posterior dorsal vertebra; (I–K) Crocodyloid cf. ''*Crocodylus*'' *depressifrons* (I) RIV.PPV 709, dorsal vertebra, (J–K) RIV.PPV 29, anterior part of left dentary; (L–O) Pleurosternid turtle *Berruchelus russelli* (L) RIV.PPV 712, peripheral, (M) RIV.PPV 713, neural, (N) RIV.PPV 714, costal and RIV.PPV 715, entoplastron plates; (P–Q) Macrobaenid turtle RIV.PPV 711, peripheral plate in (P) ventral and (Q) dorsal views; (R–S) presumably primitive turtle RIV.PPV 710, peripheral plate in (R) ventral and (S) dorsal views; (T–U) Trionychid turtle 2nd species (T) RIV.PPV 716, pleural and (U) RIV.PPV 717, neural plates; (V) ''Palaeognath'' bird RIV.PPV 718, vertebra.
doi:10.1371/journal.pone.0086229.g007

Eocene sediments, and provide further evidence suggesting a Paleocene age.

Birds. Several isolated bird bones have been discovered at Rivecourt. A large anterior dorsal vertebra, about the size of the corresponding vertebra of an ostrich, likely belongs to a ratite, possibly related to *Remiornis*, a genus known from the Thanetian of Cernay and Berru [44]. A broken zygapophysis, clearly belonging to a bird because of the cancellous texture of its bony tissue, is comparable in size and shape with zygapophyses of the giant gastornithid bird *Gastornis*, which is known in Europe from the Thanetian to the Lutetian [45]–[46], and can probably be referred to that taxon. Smaller bird bones, including a vertebra, a tibiotarsus, and a distal tarsometatarsus remain unidentified.

Discussion

Interpretation of the depositional environments and landscapes

First marine unit. The facies of the first unit (unit 0, Fig. 3) is very homogeneous all along the Petit Pâtis quarry at Rivecourt and is composed of fine sands that are very well sorted and unimodal. The occurrence of glaucony, abundant bioturbations and the dinocysts dominance among the palynomorphs in this unit suggest a marine depositional environment. This facies is common in the Thanetian sands of this area of the Paris Basin, which can reach a thickness of 15 to 30 m [18] and may correspond to the Châlons-sur-Vesles Formation or Bracheux Formation [17]. The dominance of *Spiniferites* spp. and *Operculodinium* spp. suggests shallow open marine conditions and probably reflects a marine transgressive pulse. The *Pityosporites* pollen content, dinocyst assemblage, absence of bedding, grading and ripples, absence of flint pebbles, molluscan shells and elasmobranch teeth all suggest that the depositional environment of this unit was marine, in the upper offshore of the shelf below Fair weather wave base (Fwwb) [47]–[48].

Overlying fluvial units. In the overlying units (Fig. 3) the lithology, grain size, spore and pollen assemblage, macrofossils content, and sedimentary structures observed considered all together suggest a fluvial depositional environment. Many lateral facies and grain size variations are observed, indicating a rather complex hydrographic network with meandering successive erosive channels filled by mainly sandy deposits of point-bars [47], [49]–[54]. All the beds of Unit I to IV observed on the RIVE 2 subsection are prograding towards the WSW, and the overlying ones (Unit X) of the RIVE 3 subsection towards the SSW (Fig. 3), indicating a slight change along the curve of the point-bar migration, confirmed by the slight change of direction and angle of dip of the cross-beds (Table S1a and b).

The coarser gravelly fluvial sandy sediments represent channel lag deposits that are particularly rich in fossils. Some beds with wood trunks elements, branches and many seed, fruit and flower fossils may correspond to important fluvial discharges. Among the plant remains, fossil leaves are absent, probably because they could not be preserved given the strong current velocity range. Seasonal deposition could also contribute to this sorting of plant organs.

The lignitic fine sediments that end the succession correspond to a much weaker current in the fluvial environment (low flow regime), probably resulting in the progressive abandonment of the river channel with occasional small floods yielding the fine sand of the thin sand beds and laminae, while the very fine lignitic material of the thicker beds was decanted between floods. Surprisingly at Rivecourt, no fossil leaves have been observed in this fine lignitic set, although this low-stage flow regime should have favored appropriate conditions for their preservation.

Very rapid point-bar deposition and other factors enhancing the fossilization process

In summary, although the Petit Pâtis quarry at Rivecourt does not offer particularly extensive or well exposed outcrops, several diagnostic features of the point-bar depositional environment are present: the coarse and gravelly sand of the channel lag deposit, the overall lenticular geometry, cross sets and lateral facies variation in individual sets, the horizontal bedding, large scale planar cross bedding and convolute bedding in Unit X, and finally the thinly interbedded fine sand and organic silty clay and very organic mud plug of Unit Z. Compared to the 'ideal' point-bar vertical sequence model of Plint [51], some facies are missing (reactivation surfaces, rippled sands), but those present seem sufficient to characterize a meandering river environment. At Rivecourt, four fining upward point-bar sequences are present, each one beginning by a coarse and gravelly channel lag deposit. The first and second sequences (on the RIVE 2 subsection) are thin (one meter) and probably truncated, but contain fossils (even if a granulometric bias is observed, due to the high energy of the current). The third (on the RIVE 3 subsection) and fourth sequences (on the RIVE 5, 6 and 1 subsections) are the thickest (3.5 m) and richest in plant fossils, the last fine lignitic lenticular set corresponding to the plug of the abandoned channel.

The 4 point-bar sequences preserved at Rivecourt record rapid deposition and river discharge fluctuations, with upper and lower flow regimes, suggesting possible (seasonal?) variation. The abundant and well preserved plant fossils of the fluvial sands, particularly wood trunks and branches not *in situ*, seeds and flower remains, reinforce the likelihood of a very rapid deposition, possibly in one flood per sequence, their fossilization occurring slightly later via pyritization in a reduced environment.

Interpretation of the vegetation pattern of the surrounding landscapes

In the units overlying the marine unit, the sporopollinic assemblage confirms the fluvial environment (Additional data in appendix): it is dominated by taxa from the hinterland in a subtropical climate. The low abundance of fern spores and Sparganiaceae pollen suggests that the sediments did not deposit, nor did the river carrying them across any important swamp, marsh, pond, or lake environment. The low content of *Milfordia* pollen does not indicate any generalized dry environment in the catchment either.

In addition, the vegetation pattern of the first fluvial sediment catchment corresponds to a swampy forest close to a lagoon with *Taxodium*, few Palmae and Sapotaceae. *Pinus* pollen may have been carried from distant areas by the wind or affluents, or even reworked by the river from the basal marine sand. Myricaceae and *Quercus* could occupy emerged but humid natural levees, while the hinterland was covered by a heterogeneous forest mainly composed of Juglandaceae (*Platycarya*, *Engelhardtia* and paleo *Carya*). Members of the *Tilia*, *Castanea* and *Olea* families were also present in the landscape. In the overlying X to Z units, the lagoon border flora is much reduced: few *Taxodium*, Palmae, Sapotaceae, Myricaceae and *Nyssa* are present, while the hinterland flora remains unchanged.

The macroflora assemblage seems to be consistent with the palynoflora, with a distortion probably linked to the taphonomic factors and sampling of the already studied material. Further more complete studies would give a more precise picture of this paleoflora and vegetation. In its morphology, the macrofloral element assemblage is consistent with a fluvial deposit, as observed on riverbanks in tropical and subtropical environments. The macroflora is represented by families and genera with closest living relatives in tropical and warm temperate areas. The abundance of liana remains confirms the importance of openings along the fluvial network. The numerous carbonized (fusinized) plant macro-remains highlight the occurrence of fire in the surrounding area in the fluvial catchment. The assemblage of conifers, palms and the numerous dicots listed in Table 1, shows several differences with the Le Quesnoy assemblage, especially regarding the absence of *Aulacoxylon*, a dominant species in the Paris Basin in Early Eocene palaeoenvironments [13].

The forested vegetation at Rivecourt is confirmed by the abundance of Plesiadapidae, a group of arboreal primate-like mammals. The small pleuraspidotheriid, the second most abundant mammal group, could also have been arboreal as study of the footbones of *Pleuraspidotherium* and *Orthaspidotherium* have suggested a partially arboreal locomotion [25].

Palynostratigraphy

First marine unit. Without any carbonate preserved in the sand, nannofossil or foraminiferal study is impossible; therefore, only the study of dinocysts can give biostratigraphic information.

As already mentioned above, the dinocyst assemblage from the glauconiferous sand of the basal marine unit is characterized by the rare presence of stratigraphically important species: *Alisocysta margarita*, *Deflandrea oebisfeldensis*, *Hystrichosphaeridium tubiferum, Lentinia wetzelii* and *Phthanoperidinium crenulatum*. The combined presence of these species, as well as the virtual absence of the acme of the *Areoligera gippengensis*-group, and the absence of *Apectodinium* spp. should limit the stratigraphic interval of this marine unit to a part of the upper Thanetian. The observed association corresponds best to the Danish dinocyst Zone 5 [55] and is somewhat similar to dinocyst assemblages from the Bois Gilles Sand Mb [56]–[58]. However, the Danish and Belgian associations are characterized by the rare presence of *Apectodinium* spp. In terms of nannoplankton zones, we suggest that the basal marine unit of the Rivecourt section correlates to a part of NP8/base of NP9a zones [59], corresponding to an earliest Late Thanetian age. Among the pollen assemblage of this marine unit, the presence of the taxa *Sporopollis pseudoporites*, *Stephanoporopollenites hexaradiatus* and *Tricolpopollenites hians*, restricted to the Late Thanetian [60]–[61], support this stratigraphic attribution, but does not refine it.

Overlying fluvial units. In the overlying fluvial units, *Plicapollis pseudoexcelsus* and Juglandaceae pollen grains are abundant. They are also often abundant to very abundant in the Sparnacian facies of the Paris Basin and their equivalent Tienen Formation in Belgium [60], [62]–[64]. They are much less abundant in the nearby MP7 Houdancourt "Le Quesnoy" site, where the Tricolporates, Tiliaceae and Taxodiaceae pollen grains are dominant [12]. Moreover at Rivecourt the *Normapolles* population is rather diversified and the taxa *Sporopollis pseudoporites*, *Subtriporopollenites magnoporatus magnoporatus*, *Subtriporopollenites magnoporatus tectopsilatus* and *Subtriporopollenites spissoexinus* are present, all being good stratigraphic markers of the Late Thanetian [60]–[61].

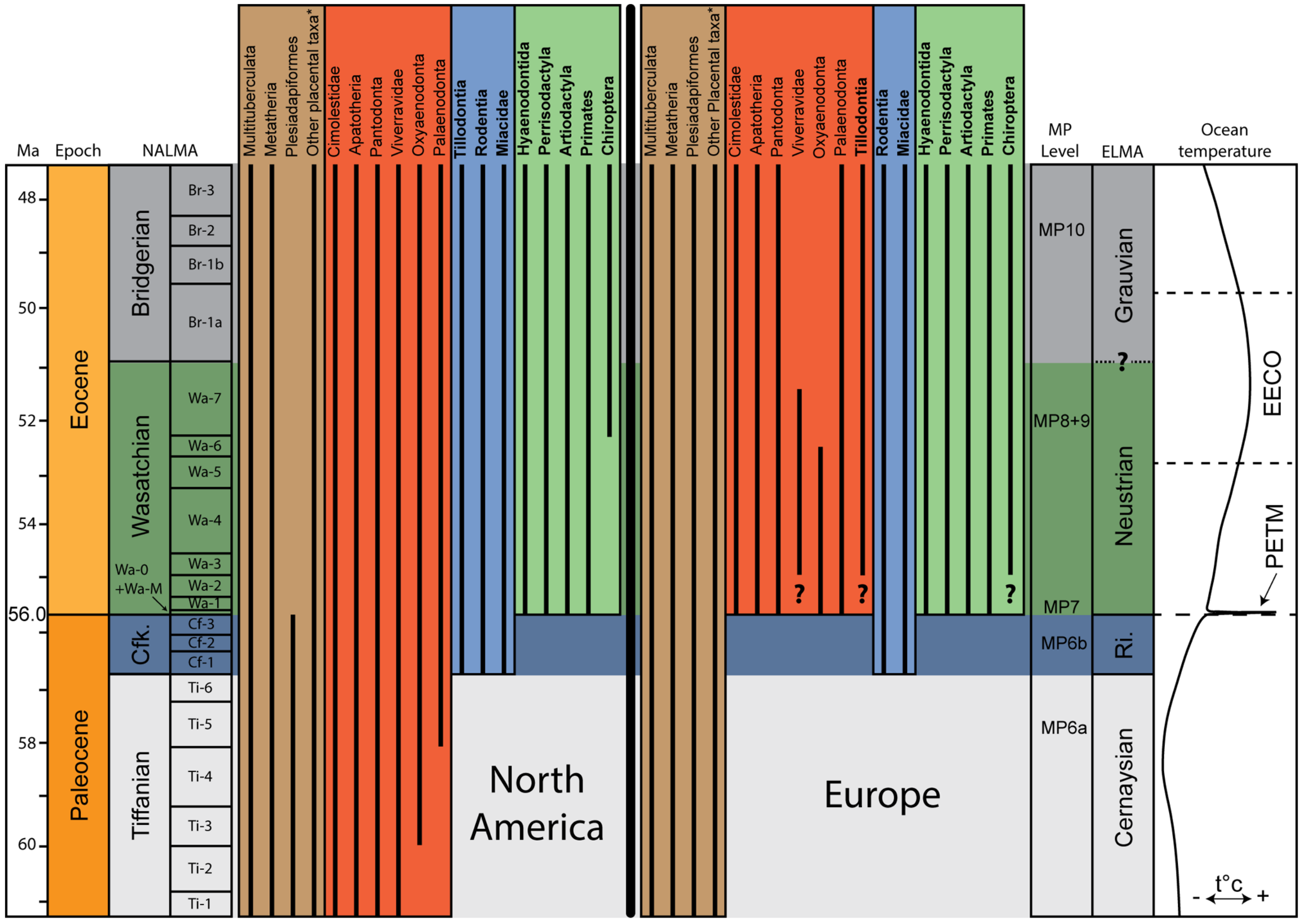

Figure 8. Stratigraphic ranges of mammalian groups and correlation of North American and European Land mammal Ages. Note the first appearances of Rodentia and "Miacidae" (blue block) in Clarkforkian and MP6b Rivecourt faunas, and that of Hyaenodontida, Perissodactyla, Artiodactyla and Primates (green block) in Wasatchian and Neustrian. These latter appearances coincide with the PETM. The brown block corresponds to typical Paleocene groups. The red block includes typical North American Paleocene mammals that are only known in Europe after Paleocene-Eocene boundary. NALMA are from Woodburne et al [99]. Ocean temperature is after Zachos et al. [100]. Other placental taxa* = Condylarthra, Lipotyphla, Procreodi, Pantolesta, and Mesonychia. Ri. = Rivecourt; ? = incertitude concerning the first appearance (mammal taxa) and/or the precise age of the fossil locality.
doi:10.1371/journal.pone.0086229.g008

Chemostratigraphy

Carbon isotope data reveals the absence of CIE onset, and $\delta^{13}C_{org}$ values ranging between −24.5‰ and −26.6‰ (Table S2, Fig. 3) are lower than values from −27 to −31‰ that would be expected for the CIE associated with the PETM in such terrestrial environments [2], [65]–[71]. The isotopic data of the Rivecourt section only enables to infer that the fluvial sediments were deposited before or after the PETM event.

Lithostratigraphy

First marine unit. The biostratigraphic data obtained for the basal marine unit of the Rivecourt section suggest an earliest Late Thanetian age. In the Paris Basin lithostratigraphic nomenclature [17], this time interval corresponds to the Châlons-sur-Vesles Formation (NP8), and the basal part of the Bracheux Formation (NP9a). However, the sedimentological features of those formations, both Late Thanetian in age, are distinctive: in this central part of the Paris Basin, the Châlons-sur-Vesles Formation sands are on average almost always glauconiferous and bioturbated, much finer and better sorted than the Bracheux Formation sands, which often have two grain size modes and include mollusks bioclasts and occasional flint pebbles. At Rivecourt, the basal marine sand is glauconiferous, bioturbated, fine, very well sorted, unimodal, and does not show any molluscan shell debris nor flint pebble. All of this evidence leads to concluding that this marine basal unit can be ascribed to the Châlons-sur-Vesles Formation.

Overlying fluvial units. Added to their position above a marine sand unit ascribed to the Châlons-sur-Vesles Formation, the chemostratigraphic and biostratigraphic data obtained on the Rivecourt site allow ascribing the fluvial lignitic sediments and their fauna and flora to the latest Thanetian. Other "Sparnacian" lignitic terrestrial sediments have been deposited before the Paleocene-Eocene boundary in the southern North Sea basins in the London Basin [69] and in the Dieppe-Hampshire Basin [66], [70]–[71]. In Avesnois (northern Paris Basin) lignitic and pyritic sands have also been deposited before the onset of the CIE indicating the P/E boundary [72]. However, none of those sites has yielded any fauna or (macro-) flora similar to those of Rivecourt. The lignitic sands of the latest Thanetian have a particular fauna and flora at Rivecourt and are stratigraphically well delineated in the more complete successions of the Cuise-La-

Motte and Le-Tillet boreholes, where they have been ascribed to the "Sables Ligniteux supérieurs". In order to distinguish this sandy and lignitic unit from other lignitic units of the earliest Eocene (the latter recording the CIE of the PETM event) much less sandy, but also very rich in fossils, a new member should be introduced in the lithostratigraphic nomenclature of the Paris Basin. This new Rivecourt Sand Member will be defined following the standard stratigraphic procedure and rules in a following article.

Mammalian biochronology and paleobiogeography

Rivecourt as close to MP6b level. The mammalian assemblage of Rivecourt corresponds to a mix between MP6 and MP7 faunas (Fig. 5). Indeed, several identified species are typical markers of the Late Thanetian (MP6), such as *Plesiadapis tricuspidens* and *Arctocyon primaevus*. The pleuraspidotheriids are known from the Late Paleocene of Cernay, Berru and Montchenot in France [25], [73] but also from the early Middle Eocene Uzunçarsidere Formation of Turkey [74] and the late Early Eocene Çeltek Formation of Turkey [26]. The new pleuraspidotheriid from Rivecourt appears more derived than *Pleuraspidotherium* and *Orthaspidotherium* from Cernay and Berru based on several characters, including more marked crests, developed hypoconulid lobe on m3, large pseudohypocone, and more lingually displaced hypoconulid on m1 and m2. The more derived state of the new species suggests a younger age than Cernay (MP6). *Landenodon woutersi*, also present in Rivecourt, is a typical marker of the earliest Eocene (MP7). The genus *Platychoerops* is present in different Early Eocene localities of Europe and has recently been described based on a dentary from the Late Paleocene of Berru with the species *P. antiquus* [75]. The identification of a typical upper incisor of *Platychoerops* in Rivecourt confirms the presence of this genus in the Paleocene. *Teilhardimys musculus* is a good marker of the Paleocene-Eocene transition as it is present both in Dormaal (MP7) and in Tremp (MP6b) in the Spanish Pyrenees [28], [76]. The species has also recently been reported from the Early Eocene of Suffolk Pebble Beds, Ferry Cliff and Kyson, and Harwich in the London Basin [27].

The mixed composition of the Rivecourt fauna is similar to the situation at Tremp (MP6b); as noted by Lopez-Martinez & Pelaez-Campomanes [76] the Spanish fauna includes both Cernaysian and Neustrian taxa. Based on this unusual mixed composition, Lopez-Martinez & Pelaez-Campomanes [76] created the level MP6b, which is intermediate between MP6 (Cernay) and MP7 (Dormaal). This unusual combination has also been found in the Romanian fauna of Jibou-Rona [77]: the Romanian fauna of Jibou interestingly includes a possible rodent among typical Paleocene taxa. Compared to Tremp and Jibou-Rona, the presence of a modern carnivoran at Rivecourt enhances the transitional aspect of the fauna of Rivecourt, which includes markers of the Late Thanetian (e.g, *Plesiadapis tricuspidens*), Paleocene-Eocene boundary (e.g., *Teilhardimys musculus*) but also the first assured representative of modern orders. Because of the presence of a rodent, Rivecourt appears also closer to the Romanian locality of Jibou-Rona than to Tremp, but conversely shares with only the Spanish localities the presence of *Teilhardimys musculus*. Finally, the three localities appear close in age, and display unusual faunas. Their composition clearly supports the originality of the MP6b level.

Characterization of the MP6b fauna and equivalence with the Clarkforkian. The Late Paleocene of North America is divided into two NALMAs, the Tiffanian and Clarkforkian, mainly known from the Fort Union Formation and Willwood Formation of Wyoming [78]. The first Early Eocene NALMA is the Wasatchian (Fig. 8). The distinction between the NALMAs is supported by first appearances of new taxa and are thus defined based on the existence of distinct mammalian faunas. The Paleocene is dominated by archaic mammals such as plesiadapids and "condylarths", while the Eocene corresponds to the earliest radiation of "modern" mammals such as primates, perissodactyls and artiodactyls.

The North American Paleocene faunas are characterized, as are the European ones, by the presence of archaic mammalian groups such as multituberculates, metatherians, plesiadapids, mesonychians, Procreodi and "condylarths" (Fig. 8, brown block). The first appearances of "modern" taxa in North America diagnose the Clarkforkian from the Tiffanian [79]. These taxa are the tillodonts (another archaic group), "miacids" and rodents (Fig. 8, blue block). The Wasatchian is characterized by the appearance of other "modern" groups: primates, perissodactyls, artiodactyls and hyaenodontidans (Fig. 8, green block). The Clarkforkian, very short in time, thus appears intermediate between the typical Paleocene and Eocene faunas.

Lopez-Martinez and Pelaez-Campomanes [76] considered that Tremp (MP6b) could be correlated to the Clarkforkian because the two faunas display low species richness and low diversity index. However, the Tremp and North American faunas do not share equivalent faunas.

The new Paleocene MP6b fauna of Rivecourt is characterized, as in North American Clarkforkian, by the presence of both "miacids" and rodents together with typical Paleocene mammals – Procreodi, "condylarths" and plesiadapids. Moreover, the fauna of Rivecourt does not include the typical Wasatchian taxa: the "modern" hyaenodontidans, primates, artiodactyls and perissodactyls (Fig. 8, green block). These taxa are indeed first known in MP7 in Europe. The discovery of "modern" mammals in MP6b locality of Rivecourt thus supports the correlation of MP6b level with the Clarkforkian.

However, a notable difference exists between the European and North American latest Paleocene faunas: the absence of tillodonts from MP6b localities. They are unknown in the MP7 reference-locality of Dormaal, but are mentioned in the faunal list of Le Quesnoy [11]; the latter locality is considered to be close to MP7 level [11], [80]. Tillodonts are thus characteristic of the Early Eocene in Europe. Moreover, it is worth noting the absence in European MP6b faunas of North American taxa such as apatotherians, cimolestids and the pantodontan *Coryphodon* – the latter being characteristic of the Clarkforkian [79]. These North American Paleocene groups are unknown in Europe until the Early Eocene (Fig. 8, red block), and appeared in Europe at the same time that "modern" hyaenodontidans, primates, artiodactyls and perissodactyls do (Fig. 8, green block).

Finally, the fauna from Rivecourt is clearly intermediate between Cernaysian and Neustrian faunas as those of the Clarkforkian are intermediate between the Tiffanian and Wasatchian. The originality of the MP6b faunas, which presently includes the localities of Tremp (Spain), Jibou (Romania) and Rivecourt (France), could justify the creation of a new ELMA. Moreover, the existence of MP6b faunas across almost the entire European continent indicates that the transition between Paleocene and Eocene faunas was a widespread event.

Paleobiogeographic implications of the MP6b migrants. As indicated above, the MP6b fauna of Rivecourt is characterized by the first appearances of two mammalian groups: rodents and "miacids" (Fig. 8, blue block). Because these two groups are unknown in previous Paleocene European faunas, they represent migrant taxa. However, unlike in North America, Rivecourt rodents and "miacids" do not appear together with

other typical Paleocene taxa such as pantodonts and cimolestids; these latter taxa only appear in Europe around the Paleocene-Eocene boundary (Fig. 8, red block). Moreover, the sole "miacid" genus recorded in Late Paleocene in North America is *Uintacyon* [81], while the Late Paleocene European "miacid" found in Rivecourt is *Vassacyon*, which is only known from Wa-0 in North America [82]. These observations suggest that the North American fauna is not the root of the MP6b migrant taxa and that the North American and European rodents and "miacids" recorded in latest Paleocene probably followed two distinct migration pathways. Asia appears a possible geographic origin for these taxa, but there are no Paleocene records for these taxa in Asia, which leaves the question open.

Conclusions

The Rivecourt terrestrial vertebrate faunal assemblage in the Paris Basin corresponds to an intermediate age between the reference level MP6 of Cernay and MP7 of Dormaal, indicating a correlation with the MP6b of the Upper part of the Tremp Formation in Spain and probably the Jibou Formation in Romania. Moreover, the presence of the earliest rodent and "miacid" carnivoran in Europe, two modern mammal groups also recorded from the latest Paleocene of North America, makes the Rivecourt assemblage a direct equivalent to the Clarkforkian North American Land Mammal Age. The discovery of this new European Land Mammal Age constitutes important progress as it fills a gap in the biochronology of the Paleogene that has been debated for decades.

The new fossils have been found in fluvial sediments of the basal Sparnacian facies of the north-central part of the Paris Basin, in the Rivecourt Sand Member, at the base of the Mortemer Formation, and below the CIE onset marking the Paleocene-Eocene boundary.

Rivecourt represents a meandering river depositional environment with a megathermal landscape vegetation pattern surrounding the locality. Fluvial sediments were deposited very rapidly, probably during seasonal flood episodes. Mammals are only moderately diverse and not particularly abundant whereas turtles and champsosaurs are especially abundant.

Materials and Methods

Field work and material collected

Fieldwork in Rivecourt has been possible thanks to permission and logistic support of the Lafarge Granulats Company. All necessary permits were obtained for the described study, which complied with all relevant regulations. Sedimentological material is stored in BRGM (Orléans, France). Palynological material is stored in BRGM (Orléans, France) and at the University of Liège (Belgium). Fossil flora material is stored in the Musée Antoine Vivenel (Compiègne, France) under the collection name RIV.PPB (Rivecourt, Petit Pâtis, Botany). Fossil fauna material is stored in the Musée Antoine Vivenel (Compiègne, France) under the collection name RIV.PPV (Rivecourt, Petit Pâtis, Vertebrate).

The Rivecourt "Petit Pâtis" locality (N 49°20′10″, E 02°44′09″) is part of a quarry operated by the Lafarge Company for its aggregate extraction activity. The Oise Quaternary alluvium deposits mined in that quarry overlie "Sparnacian" fluvial lignitic and pyrite rich sand units and/or Thanetian marine sands (Figs. 1 and 2).

The geological study of the succession was carried out in August-September 2009 on 6 subsections in the quarry, after a powered pump dropped the water table level, but almost a third of the quarry surface remained below the water. Six trenches (RIVE 1 to RIVE 6) were dug in order to study the succession (red lines on Fig. 2).

Field observations were performed on each subsection; sedimentological logs were developed in order to describe rock units including their geometric and stratigraphic relationships, lithological content and sedimentary structures (Fig. 3). Panoramic sketches were drawn on each trench dug and illustrated by numerous photos in order to follow the contacts between units and the varying contents of each one. Detailed observations and photos were also taken in order to document each unit facies and facies variations. Direction and angle of dip of the cross-beds were measured where 3D observation was possible (Table S1a). The direction of wood debris was also measured where it was possible (Table S1b). 115 samples were collected in the field, then immediately dried at the laboratory for further petrographic, grain size, isotopic and palynologic analyses.

Grain size

Grain size analysis was performed at the BRGM laboratories on 20 sand samples selected as being representative of all the sandy lithofacies identified. Around 200 g of each sample were sieved in column. The sieving for the smaller fraction (20 to 80 μm) was carried out with water flux. The upper part was dried, and then sieved between 4000 μm and 80 μm. Each dried sieved residue was weighed. These weights were analyzed by Folk and Ward's [83] and moment methods.

Carbon isotope data

The carbon-isotope ratios of bulk organic matter (Dispersed Organic Carbon [DOC] or Particulate Organic Carbon [POC]) were measured on 17 samples spanning 9 m of the succession in the fluvial sands. The samples were prepared at the University of Namur (see [84] for details) and the isotope measurements were performed at the Parma University. Bulk sediment samples of about 40 g each were first dried and then cleaned, removing surface oxidation to exclude potential sources of degraded organic matter. Even if not carbonated, samples were also powdered and treated with HCl 25% for at least 1 hour to be sure carbonates were removed. Soluble salts were removed by repetitive centrifuging (4000 revolutions per minute) until the neutral solution was obtained. Finally the residue was dried at 35°C and powdered again. Fractions of each resulting powder were measured with a standard LECO carbon analyzer (CS-200) to determine total organic carbon (TOC). Quantities required for analysis (between 0.07 and 25.1 mg) were calculated on the basis of the TOC values. Each sample was weighed into tin capsules and rolled into balls for continuous flow combustion and isotopic analysis using a Carlo Erba EA1110 elemental analyzer coupled to a mass spectrometer (Thermo Finnigan Delta Plus XP). The analyses were performed combusting the samples at 1025°C. Measured isotopic compositions were calibrated with the inter-laboratory international standards: sucrose IAEA-CH-6, oil NBS-22 and graphite USGS-24. TOC contents were checked by comparing to a laboratory standard (urea). Both standards (0.025 to 0.2 mg respectively in a purified tin cup) were measured repeatedly between each set of twenty samples. Organic ^{13}C values (Fig. 3, Table S2) are reported as a proportion of ^{12}C in $\delta^{13}C$ notation normalized to the international PDB standard (VPDB, Vienna Peedee Belemnite). Two distinct measurements were made for almost all samples. The overall precision of analyses is within 0.2‰ (1σ).

Palynology

Palynologic preparation was performed on six samples in the main facies among the 115 samples collected: one from the glauconiferous sand of the marine first unit, then five samples from the overlying fluvial lignitic units. Two palynologic slides per sample were prepared in the British Geological Survey and Liège University laboratories according to standard preparation procedure: dissolution of carbonates and silicates by HCl and HF acid digestion, sieving between 106 and 10 µm, neutralization with distilled water and centrifugation, then mounting of the remaining residues on the slides. A slight acetolysis was performed for the samples richer in organic matter.

All the pollen grains and spores were counted within each slide at the Liège University. The pollen and spores morphologic and taxonomic nomenclature follows Pflug [85]–[86], Krutzsch [87], Roche [60] and Krutzsch & Vanhoorne [88]. For a single productive sample (RIVE 2-0) terrestrial and marine palynomorphs were firstly counted together, then dinoflagellate cysts, acritarchs and other 'miscellaneous' algae were counted separately until ~300 specimens at the Russian Academy of Sciences. Subsequently, remaining materials were scanned for rare dinocyst taxa. The dinoflagellate cyst nomenclature follows Fensome & Williams [89].

Macroflora

Almost all specimens were collected by screen washing of fossiliferous sediments on meshes of 5, 2, and 1 mm and density separated from the denser mineralized material in water. The obtained lignite was washed with tap water and dried in a ventilated oven at 45°C. Sorting was done under binocular microscopes. The specimens are kept in plastic boxes with renewed silica gel. They were observed under a Wild M3Z binocular microscope and imaged by a Nikon D300 camera.

Vertebrate paleontology

With the exception of rare large vertebrate remains, most of vertebrate specimens were collected by screen washing of fossiliferous sediments on meshes of 5, 2, and 1 mm. Sorting was done under binocular microscope. All the specimens were treated at RBINS laboratories with the binder Degalan P24, a polymer based on methyl methacrylate and n-butyl methacrylate in order to consolidate and stop the oxidation. Photography of small specimens was done at the RBINS with an environmental scanning electronic microscope FEI Quanta 200. Specimens larger than 10 mm were photographed with a digital camera after covering with ammonium chloride in order to better see the surface structures, because all the fossil material is of dark brown to black color.

Patient Privacy and Informed Consent for Publication

The subject of the photograph in Figure 3 has given written informed consent, as outlined in the PLOS consent form, to publication of their photograph.

Supporting Information

Figure S1 Fieldwork in central part of the Petit Pâtis Quarry in Rivecourt (summer 2012). North is at right, south at left and the RD200 road on the backside. The mechanical shovel is just behind section Rive 2.
(TIF)

Figure S2 Palynology of the Petit Pâtis Quarry in Rivecourt. Sedimentologic log of the composite section (same legend as for Fig. 3) and palynology abundance curves. (A) Palynomorphs content, (B) Main pollen taxa among which *Normapolles* and Juglandaceae. (C) Gymnosperms, lime and pre-Quercus pollen grains and warm, humid/dry environment indicators.
(TIF)

Table S1 Sedimentological (paleocurrents) compass measurements on the Petit Pâtis Quarry in Rivecourt. (A) Direction and angle of dip of the cross-beds from the RIVE 2 and RIVE 3 subsections; (B) Direction and size of the wood debris from the RIVE 2 subsection.
(DOC)

Table S2 Chemostratigraphic data of the Petit Pâtis Quarry composite section in Rivecourt. Lithology, elevation (m), samples number, TOC (%) and $\delta^{13}C_{org}$ values (average of 2 measurements) of all the samples analyzed.
(DOC)

Table S3 Diversity of dinoflagellate cysts of the Petit Pâtis Quarry composite section in Rivecourt.
(DOC)

Acknowledgments

Fieldwork in Rivecourt has been possible thanks to permission and logistic support of Cédric de Collasson, Patrick Lobjois, Sandra Rimey and Rémi Maïa (Lafarge Granulats Company).

Special thanks are addressed to Catherine Truffert (Research Division of the BRGM) for encouraging and supporting research on early Paleogene stratigraphy of the Paris Basin and Eric Bas (CPIE) for promoting research on paleomammalogy in the Oise region. We thank Pieter Missiaen and Richard Smith (RBINS) for screen washing assistance in the field, Nathalie Van Hoey (RBINS) and Renaud Vacant (MNHN) for sorting the vertebrate remains from the screen washed residue, Fabrice Vanderlinden (RBINS) for preparing all the vertebrate specimens, J-C Paicheler for the geological cross section, slightly modified in the Fig. 1, Gautier Folgoas and Romain Ganet (Lassale Beauvais) for a first version of the geological map of the Petit Pâtis quarry, Maud André (BRGM) for field work assistance, Hubert Haas and Aurélien Leynet (BRGM) for their help with the grain size analyses and statistical processing, Fabrice Moreau (Compagnie générale de Géophysique) and Richard Smith (RBINS) for help with the elasmobranch identification. We are grateful to editor Richard J. Butler and reviewer Douglas M. Boyer for their comments and suggestions that greatly improved our manuscript, and Gregg F. Gunnell for linguistic revision and helpful suggestions.

Author Contributions

Conceived and designed the experiments: TS FQ. Performed the experiments: TS FQ GDP DDF GM EDB FS AF JC CD CG FM JP EB. Analyzed the data: TS FQ DDF GM EDB FS AF AB JC CD CG AI JM ER JYS RT HT JY EB. Wrote the paper: TS FQ DDF EDB FS AF JC JM JYS HT EB.

References

1. Russell DE (1964) Les mammifères paléocènes d'Europe. Mémoires du Muséum National d'Histoire Naturelle, Série C 8: 1–324.
2. Smith T, Rose KD, Gingerich PD (2006) Rapid Asia-Europe-North America geographic dispersal of earliest Eocene primate *Teilhardina* during the Paleocene-Eocene Thermal Maximum. Proc Natl Acad Sci USA 103 (30): 11223–11227.
3. Teilhard de Chardin P (1927) Les Mammifères de l'Eocène inférieur de la Belgique. Mémoires du Musée Royale d'Histoire Naturelle de Belgique 6: 1–33.
4. Smith T, Smith R (1996) Synthèse des données actuelles sur les vertébrés de la transition Paléocène–Eocène de Dormaal (Belgique). Bulletin de la Société belge de Géologie 104: 119–131.

5. Smith T (2000) Mammals from the Paleocene-Eocene transition in Belgium (Tienen Formation, MP7): Palaeobiogeographical and biostratigraphical implications. In Schmitz, B., Sundquist, B. & Andreansson, F.P. (eds.): Early Paleogene warm climates and biosphere dynamics. GFF 122: 148–149.
6. Schmidt-Kittler N Ed (1987) European Reference Levels and correlation tables. Münchner Geowissenshaften Abhandlungen A 10: 13–31.
7. BiochroM'97 (1997) Synthèse et tableaux de corrélations. In Aguilar, J.P., Legendre, S. & Michaux, J. (eds.): Actes du Congrès BiochroM'97. Mémoires et Travaux de l'EPHE, Institut de Montpellier 21, 769–805.
8. Magioncalda R, Dupuis C, Smith T, Steurbaut E, Gingerich PD (2004) Paleocene-Eocene carbon isotope excursion in organic carbon and pedogenic carbonate: Direct comparison in a continental stratigraphic section. Geology 32 (7): 553–556.
9. Yans J, Strait SG, Smith T, Dupuis C, Steurbaut E, et al. (2006) High-resolution carbon isotope stratigraphy and mammalian faunal change at the Paleocene-Eocene boundary in the Honeycombs area of the Southern Bighorn Basin, Wyoming. American Journal of Science 306: 712–735.
10. De Ploëg G, Dutheil D, Gheerbrant E, Godinot M, Jossang A, et al. (1998) Un nouveau gisement paléontologique à la base de l'Eocène dans la région de Creil (Oise). Strata 9: 108–110.
11. Nel A, de Ploëg G, Dejax J, Dutheil D, De Franceschi D, et al. (1999) Un gisement sparnacien exceptionnel à plantes, arthropodes et vertébrés (Eocène basal, MP7): Le Quesnoy (Oise, France). C R Acad Sci Paris (Sci Terre Planètes) 329: 65–72.
12. Cavagnetto C (2000) La palynoflore d'un gisement d'ambre de l'Eocène basal du Bassin Parisien (Le Quesnoy, France). Palaeontographica Abt B 255 (4–6): 147–171.
13. De Franceschi D, De Ploëg G (2003) Origine de l'ambre des faciès sparnaciens (Éocène inférieur) du Bassin de Paris: le bois de l'arbre producteur. Geodiversitas 25 (4): 633–647.
14. Jacques FMB, De Franceschi D (2005) Endocarps of Menispermaceae from Le Quesnoy outcrop (Sparnacian facies, Lower Eocene, Paris Basin). Review of Palaeobotany and Palynology 135: 61–70.
15. Cavelier C (1968) Coupes détaillées des sondages exécutés dans le Paléogène du Bassin de Paris à Chaignes (Eure), Montjavoult (Oise), Cires-lès-Mello - Le Tillet (Oise), Ludes (Marne). Mémoire BRGM 59: 13–52.
16. Blondeau A, Fraisse C, Pomerol B, Pomerol C, Raffin C, et al. (1976) Geological Map of France (1/50 000), sheet 81 (Montdidier), Editions BRGM, Orléans.
17. Aubry M-P, Thiry M, Dupuis C, Berggren WA (2005) The Sparnacian deposits of the Paris Basin: A lithostratigraphic classification. Stratigraphy 2-1: 65–100.
18. Rouvillois A (1960) Le Thanétien du Bassin de Paris (Etude hydrogéologique et micropaléontologique). Mémoire du Museum d'Histoire Naturelle de Paris, Sciences de la Terre, VIII, 153 p.
19. Feugueur L (1963) L'Yprésien du Bassin de Paris. Essai de monographie stratigraphique. Mémoire pour servir à l'explication de la Carte géologique de France 568 p.
20. Graves L (1847) Essai sur la topographie géognostique du Département de l'Oise. Imprimerie Desjardins, Beauvais. 804 p.
21. Aubry M-P, Ouda K, Dupuis C, Berggren WA, Van Couvering JA (2007) The Global Standard Stratotype-section and Point (GSSP) for the base of the Eocene Series in the Dababiya section (Egypt). Episodes 30(4): 271–286.
22. Gingerich PD (1976) Cranial Anatomy and Evolution of Early Tertiary Plesiadapidae (Mammalia, Primates). University of Michigan Papers on Paleontology 15. 140 p.
23. Godinot M (1980) La variabilité morphologique dentaire du *Landenodon* (Condylarthra) de Dormaal (Eocène, Belgique). Palaeovertebrata, Mémoire Jubilaire R. Lavocat: 167–196.
24. Russell DE (1980) Sur les condylarthres cernaysiens *Tricuspiodons* et *Landenodon* (Paléocène supérieur de France). Palaeovertebrata, Mémoire Jubilaire R Lavocat: 127–166.
25. Ladevèze S, Missiaen P., Smith T (2010) First skull of *Orthaspidotherium edwardsi* (Mammalia, "Condylarthra") from the late Paleocene of Berru (France) and phylogenetic affinities of the enigmatic European family Pleuraspidotheriidae. Journal of Vertebrate Paleontology 30: 1559–1578.
26. Métais G, Gheerbrant E, Sen S (2012) Re-interpretation of the genus *Parabunodon* (Ypresian, Turkey): implications for the evolution and distribution of pleuraspidotheriid mammals. Palaeobiodiversity and Palaeoenvironments 92: 477–486.
27. Hooker JJ, Russell DE (2012) Early Palaeogene Louisinidae (Macroscelidea, Mammalia), their relationships and north European diversity. Zoological Journal of the Linnean Society 164: 856–936.
28. Tabuce R, Telles Antunes M, Smith R, Smith T (2006) Dental variability and tarsal morphology of the European Paleocene/Eocene "condylarth" mammal Microhyus. Acta Palaeontologica Polonica 51: 37–52.
29. Solé F, Smith R, Coillot T, De Bast E, Smith T (in press) Dental and tarsal anatomy of "*Miacis*" *latouri* and a phylogenetic analysis of the earliest carnivoraforms (Mammalia, Carnivoramorpha). Journal of Vertebrate Paleontology.
30. Gagnaison C, Prieur J, de Ploëg G (2009) Le gisement de vertébrés thanétiens (Paléocène supérieur, MP6) du Petit Pâtis près de Creil (Oise, France). Bulletin d'information du Bassin de Paris 46 (2): 3–11.
31. Sanchiz B (1998) Encyclopedia of Paleoherpetology. Gustav Fischer Verlag, New York. 275 p.
32. Herre W (1950) Schwanzlurche aus dem Paleocän von Walbeck. Zoolegischer Anzeiger (Klatt-Festschrift) 145: 286–301.
33. Folie A, Smith R, Smith T (2013) New amphisbaenian lizards from the Early Paleogene of Europe and their implication in the early evolution of modern amphisbaenians. Geologica Belgica 16 (4): 227–235.
34. Estes R (1983) *Encyclopedia of Paleoherpetology*, Part 10A: Sauria terrestria, Amphisbaenia, New York. 249 p.
35. Estes R, De Queiroz K, Gauthier J (1988) *Phylogenetic relationships within Squamata. In*: Estes R. & Pregill G. (eds), Phylogenetic relationships of the lizard families, Stanford, California. 119–281.
36. Rage J-C (2001) Fossil snakes from the Palaeocene of São José de Itaboraí, Brazil. Part II Boidae. Palaeovertebrata 30: 111–150.
37. Sigogneau-Russell D, de Heinzelin J (1979) Les Champsosaures européens: Mise au point sur le Champsosaure d'Erquelinnes (Landénien inférieur, Belgique), avec introduction stratigraphique. Annales de Paléontologie, Vertébrés 65 (2): 93–154.
38. Delfino M, Smith T (2012) Reappraisal of the morphology and phylogenetic relationships of the middle Eocene alligatoroid *Diplocynodon deponiae* (Frey, Laemmert and Riess, 1987) based on a 3D-prepared specimen. Journal of Vertebrate Paleontology, 32: 1358–1369.
39. Delfino M, Smith T (2009) A reassessment of the morphology and taxonomic status of *'Crocodylus' depressifrons* Blainville, 1855 (Crocodylia, Crcodyloidea) based on the early Eocene remains from Belgium. Zoological Journal of the Linnean Society 156: 140–167.
40. Broin F de (1977) Contribution à l'étude des Chéloniens. Chéloniens continentaux du Crétacé et du Tertiaire de France. Mémoires du Muséum national d'Histoire naturelle. Nouvelle série. Série C, tome 33. 365 p.
41. Vitek NS (2012) Giant fossil soft-shelled turtles of North America. Palaeontologia Electronica, 15 (1): 43p.
42. Pérez-García A (2012) *Berruchelus russelli*, gen. et sp. nov., A Paracryptodiran Turtle from the Cenozoic of Europe. Journal of Vertebrate Paleontology 32: 545–556.
43. Sukhanov VB (2000) Mesozoic turtles of Middle and Central Asia. In: The Age of Dinosaurs in Russia and Mongolia, Benton MJ, Shishkin MA, Unwin DM, Kurochkin EN (eds), Cambridge University Press, Cambridge 309–367.
44. Martin LD (1992) The status of the Late Paleocene birds *Gastornis* and *Remiornis*. Nat Hist Mus Los Angeles County Sc Ser 36: 97–108.
45. Buffetaut E (2002) Giant birds at the Cretaceous-Tertiary boundary: extinction or survival ? Geol Soc America Spec Paper 356: 303–306.
46. Mayr G (2009) Paleogene fossil birds. Springer, Berlin. 262 p.
47. Reineck HE, Singh IB (1980) Depositional sedimentary environments, with reference to terrigenous clastics. Springer Verlag, second revised and updated edition, Berlin, 551 pp.
48. Johnson HD, Baldwin CT (1996) Shallow clastic seas. *In:* Sedimentary environments: processes, facies and stratigraphy, READING HG Ed 7: 232–280.
49. Allen JRL (1964) Studies in fluviatile sedimentation. Six cyclothems from the Old Red Sandstone, Anglo-Welsh Basin. Sedimentology 3: 163–198.
50. Schumm SA (1977) The fluvial system. Wiley & Sons, New York. 338 p.
51. Plint AG (1983) Facies, environments and sedimentary cycles in the Middle Eocene, Bracklesham Formation of the Hampshire Basin: evidence for global sea-level changes? Sedimentology 30: 625–653.
52. Plint AG (1988) Sedimentology of the Eocene strata exposed between Poole Harbour and High Cliff, Dorset, UK. Tertiary Research 10(3): 107–145.
53. Bridge JS, Smith ND, Trent F, Gabel SL, Bernstein P (1986) Sedimentology and morphology of a low sinuosity river: Calamus River, Nebraska Sand Hills. Sedimentology 33:851–870.
54. Collinson JD (1996) Alluvial sediments. *In:* Sedimentary environments: processes, facies and stratigraphy, READING HG Ed. 3: 37–82.
55. Heilmann-Clausen C (1985) Dinoflagellate stratigraphy of the uppermost Danian to Ypresian in the Viborg 1 borehole, central Jylland, Denmark. Danm Geol UndersØg (A) 7: 69 pp.
56. De Coninck J, De Decker M, De Heinzelin J, Willems W (1981) L'Age des faunes d'Erquelinnes. Bull Soc belge Géol 90 (2): 121–154.
57. Steurbaut E (1998) High-resolution holostratigraphy of middle Paleocene to early Eocene strata in Belgium and adjacent areas. Palaeontographica Abt A 247: 91–156.
58. Knox RW O'B, Bosch A, Rasmussen ES, Heilmann-Clausen C, Hiss M, et al. (2010) Cenozoic. *In:* Petroleum Geological Atlas of the Southern Permian Basin Area, Doornenbal JC, Stvenson AG (Eds), EAGE Publications bv 12: 210–223.
59. Martini E (1971) Standard Tertiary and Quaternary calcareous nannoplankton zonation. *In*: Farinacci (Ed.), Proc. Second Conf. Planktonic Microfossils, Roma, 1970, Vol. 2. Tecnoscienza, Roma, 739–785.
60. Roche E (1973) Etude des Sporomorphes du Landénien de Belgique et de quelques gisements du Sparnacien français. Mém Servir Explic Cart Géol Minist Belg 13: 13–121.
61. Roche E, Dupuis C, Quesnel F (2009) Tentative high resolution palynostratigraphy of the Early Eocene in the southern North Sea Basin, In: Climatic and Biotic Events of the Paleogene, Strong CP, Crouch EM, Hollis C (Eds), GNS Science Miscellaneous Series 16: 122.
62. Gruas-Cavagnetto C (1968) Étude palynologique de divers gisements du Sparnacien du bassin de Paris. Mém. Soc. Géol. France 110: 1–144.

63. Chateauneuf JJ, Gruas-Cavagnetto C (1968) Etude palynologique du Paléogène de quatre sondages du bassin parisien (Chaignes, Montjavoult, Le Tillet, Ludes). Mémoires du BRGM 59: 113–162.
64. Gruas-Cavagnetto C (1976) Etude palynologique du sondage de Cuise-la-Motte (Oise). Bull Inf Géol Bass Paris 13(2): 11–23.
65. Stott LD, Sinha A, Thiry M, Aubry M-P, Berggren WA (1996) The transfer of ^{12}C changes from the ocean to the terrestrial biosphere across the Paleocene/Eocene boundary: criteria for terrestrial-marine correlation. *In:* Correlalion of the early Paleogene in Northwest Europe, Knox RW O'B, Corfield R, Dunay RE (eds), Geological Society Special Publication, London 101: 381–399.
66. Magioncalda R, Dupuis C, Blamart D, Fairon-Demaret M, Perreau M, et al. (2001) L'excursion isotopique du carbone organique ($\delta^{13}C_{org}$) dans les paléoenvironnements continentaux de l'intervalle Paleocène/Eocène de Varengeville (Haute-Normandie). Bull Soc Géol France 172 (3): 349–358.
67. Steurbaut E, Magioncalda R, Dupuis C, Van Simaeys S, Roche E, et al. (2003) Palynology, paleoenvironments, and organic carbon isotope evolution in lagoonal Paleocene-Eocene boundary settings in North Belgium, In: Causes and Consequences of Globally Warm Climates in the Early Paleogene, Wing SL, Gingerich PD, Schmitz B, Thomas E (eds), Boulder, Colorado, Geological Society of America Special Paper 369: 291–317.
68. Thiry M, Aubry MP, Dupuis C, Sinha A, Stott LD, et al. (2006) The Sparnacian deposits of the Paris Basin: δ^{13}C isotope stratigraphy. Stratigraphy 3 (2): 119–138.
69. Collinson ME, Steart DC, Scott AC, Glasspool IJ, Hooker JJ (2007) Episodic fire, runoff and deposition at the Palaeocene–Eocene boundary. Journal of the Geological Society, London 164: 87–97.
70. Smith T, Dupuis C, Folie A, Quesnel F, Storme JY, et al. (2011) A new terrestrial vertebrate site just after the Paleocene–Eocene boundary in the Mortemer Formation of Upper Normandy, France. CR Palevol 10: 11–20.
71. Storme J-Y, Dupuis C, Schnyder J, Quesnel F, Garel S, et al. (2012) Cycles of humid-dry climate conditions around the P/E boundary: new stable isotope data from terrestrial organic matter in Vasterival section (NW France). Terra Nova 24: 114–122.
72. Quesnel F (2006) Méso-Cénozoïque, Crétacé et Cénozoïque, 3 geological maps and 87 p of explanatory notes, *In:* Lacquement, F., Quesnel, F., Mansy, J.L., Moulouel, H., Vinchon, C., Gateau, C. et coll., 2006, La Géologie du territoire de l'Avesnois, Système d'information géologique, DVD, *BRGM/RP-55465-FR*.
73. Jehle M, Godinot M, Delsate D, Phélizon A, Pellouin J-L (2012) A new late Paleocene micromammal fauna from Montchenot (Paris Basin). Preliminary results. Palaeobiodiversity and Palaeoenvironments. DOI 10.1007/s12549-012-0100-x.
74. Maas MC, Thewissen JGM, Sen S, Kazanci N, Kappelman J (2001). Enigmatic new ungulates from the early middle Eocene of Central Anatolia, Turkey. Journal of Vertebrate Paleontology 21(3): 578–590.
75. Boyer DM, Costeur L, Lipman Y (2012) Earliest Record of *Platychoerops* (Primates, Plesiadapidae), A New Species From Mouras Quarry, Mont de Berru, France. American Journal of Physical Anthropology 149: 329–346.
76. López-Martínez N, Peláez-Campomanes P (1999) New mammals from South-central Pyrenees (Tremp Formation, Spain) and their bearing on Late Paleocene marine-continental correlations. Bulletin Société Géologique de France 170: 681–686.
77. Gheerbrant E, Codrea V, Hosu Al, Sen S, Guernet C, Lapparent de Broin Fr, Riveline J (1999) Découverte de vertébrés dans les Calcaires de Rona (Thanétien ou Sparnacien), Transylvanie, Roumanie: les plus anciens mammifères cénozoiques d'Europe Orientale. Eclogae Geologie Helvetiae, 92: 517–535.
78. Gingerich PD, Clyde WC (2001) Overview of Mammalian Biostratigraphy in the Paleocene-Eocene Fort Union and Willwood Formations of the Bighorn and Clarks Fork Basins. University of Michigan Papers on Paleontology 33: 1–14.
79. Rose KD (1981) The Clarkforkian land-mammal age and mammalian faunal composition across the Paleocene-Eocene boundary. Papers on Paleontology 26: 1–115.
80. Solé F, Gheerbrant E, Godinot M (2011) New data on the Oxyaenidae from the Early Eocene of Europe; biostratigraphic, paleobiogeographic and paleoecologic implications. Palaeontologica Electronica 14, 2: 41p. org/2011_2/258/index.html
81. Gingerich PD (1983) Systematics of Early Eocene Miacidae (Mammalia, Carnivora) in the Clark's Fork Basin, Wyoming. Contributions from the Museum of Paleontology, 26: 187–225.
82. Heinrich RE, Strait SG, Houde P (2008) Earliest Eocene Miacidae (Mammalia: Carnivora) from northwestern Wyoming. Journal of Paleontology 82: 154–162.
83. Folk RL, Ward WC (1957) Brazos river bar: a study in the significance of grain size parameters. J Sediment Petrol 27: 3–26.
84. Yans J, Gerards T, Gerrienne P, Spagna P, Dejax J, et al. (2010) Carbon-isotope of fossil wood and dispersed organic matter from the terrestrial Wealden facies of Hautrage (Mons basin, Belgium). Palaeogeography, Palaeoclimatology, Palaeoecology 291: 85–105.
85. Pflug H (1952) Palynologie und Stratigraphie des eozänen Braunkohlen von Helmstedt. Paläeont Z 26: 112–137.
86. Pflug H (1953) Zur Entstehung und Entwicklung des angiospermiden Pollens in der Erdgeschichte. Palaeontographica B95 (4–6): 60–171.
87. Krutzsch W (1958) Sporen- und Pollengruppen aus der Oberkreide und dem Tertiär Mitteleuropas und ihre stratigraphische Verteilung. Zeitschrilt für angewandte Geologie 11–12: 509–548.
88. Krutzsch W, Van Hoorne R (1977) Die Pollenflora von Epinois und Loksbergen in Belgien, Palaeontographica Abt B 163 (1–4): 1–110.
89. Fensom RA, Williams GL (2004) The Lentin and Williams Index of Fossil Dinoflagellates 2004 Edition. AASP Contribution Series 42, 909 p.
90. Blondeau A (1970) Geological Map of France (1/50 000), sheet 104 (Compiègne), Editions BRGM, Orléans.
91. Solau J-L, Cruciani P-M, Maucorps J, Pomerol C (1974) Geological Map of France (1/50 000), sheet 105 (Attichy), Editions BRGM, Orléans.
92. Guérin B, Salin R, Pomerol C (1975) Geological Map of France (1/50 000), sheet 129 (Villers-Cotterêt), Editions BRGM, Orléans.
93. Pomerol C, Cavelier C, Blondeau A, Afchain C, Dubois R, et al. (1967) Geological Map of France (1/50 000), sheet 128 (Senlis), Editions BRGM, Orléans.
94. Cavelier C (1967) Geological Map of France (1/50 000), sheet 127 (Creil), Editions BRGM, Orléans.
95. Blondeau A, Cavelier C, Monciardini C (1970) Geological Map of France (1/50 000), sheet 103 (Clermont), Editions BRGM, Orléans.
96. Blondeau A, Froehlich F, Pomerol B, Pomerol C (1978) Geological Map of France (1/50 000), sheet 80 (Saint-Just-en-Chaussée), Editions BRGM, Orléans.
97. Blondeau A, Gruas-Cavagnetto C, Le Calvez Y, Lézaud L (1976) Etude paléontologique du sondage de Cuise (Oise). Bull Inf Géol Bass Paris 13: 3–31.
98. Solau J-L, Pomerol B, Maucorps J, Pomerol C, Bourse C, et al. (1976) Geological Map of France (1/50 000), sheet 82 (Chauny), Editions BRGM, Orléans.
99. Woodburne MO, Gunnell GF, Stucky RK (2009) Land mammal faunas of North America: Rise and fall during the Early Eocene Climatic Optimum. Denver Mus Nat Sci Ann. 1: 1–80.
100. Zachos J, Pagani M, Sloan L, Thomas E, Billups K (2001) Trends, rhythms, and aberrations in global climate 65 Ma to present. Science 292: 686–693.
